# Vascular-related proteomic signatures in COPD with suspected pulmonary hypertension as predictors of FEV₁ impairment

**DOI:** 10.1186/s12931-026-03622-5

**Published:** 2026-03-28

**Authors:** Khushboo Goel, Ryen Ormesher, Katherine A. Pratte, Yue Wang, Koichi Nishino, Craig P. Hersh, Katerina Kechris, Russell P. Bowler, Irina Petrache

**Affiliations:** 1 Department of Medicine, Division of Pulmonary and Critical Care Medicine, Cedars- Sinai Medical Center, Los Angeles, CA USA; 2 Department of Medicine, Division of Pulmonary Critical Care and Sleep Medicine, National Jewish Health, Denver, CO USA; 3 Department of Medicine, Division of Pulmonary Sciences and Critical Care Medicine, University of Colorado, Aurora, CO USA; 4 Department of Biostatistics, National Jewish Health, Denver, CO USA; 5 Department of Biostatistics and Informatics, University of Colorado, Aurora, CO USA; 6 Department of Medicine, Channing Division of Network Medicine, Brigham and Women’s Hospital, Boston, MA USA; 7 Department of Genomic Sciences and Systems Biology, Cleveland Clinic, Cleveland, OH USA; 8 Pulmonary, Critical Care and Sleep Medicine, COPD Research & Medicine, National Jewish Health, 1400 Jackson Street, K803, Denver, CO 80206 USA

**Keywords:** COPD-associated Pulmonary Hypertension (PH), Group III PH, MicroRNA-126 (miR-126), Endothelial Dysfunction, Mediation Analysis

## Abstract

**Background:**

The development of pulmonary hypertension (PH) is a serious complication of chronic obstructive pulmonary disease (COPD). Despite advances in characterizing pulmonary vascular remodeling in COPD-PH, the lack of targeted therapies limits the routine use of gold-standard invasive diagnostics, highlighting the need for novel biomarkers. The pulmonary vascular endothelium is central to the pathogenesis of both PH and COPD. Since most endothelium-derived modulators of vascular tone and remodeling are targets of endothelial-enriched microRNA-126 (miR-126), a master vascular regulator that is suppressed in COPD, these and related ‘angiocentric molecules’ may be promising biomarkers for COPD-PH.

**Research Goal:**

To identify angiocentric proteins elevated in individuals with suspected COPD-PH, defined by a pulmonary artery-to-aorta ratio (PA/A) > 1 on thoracic CT, and to evaluate if they are significantly associated with the severity of airflow limitation (FEV₁).

**Study Design:**

We analyzed plasma proteomic profiles from 1,056 COPDGene Phase-1 participants. Using PA/A > 1 as the outcome, we identified differentially abundant angiocentric proteins. We then assessed the abundance of angiocentric proteins in those with severe airflow obstruction (FEV₁ <50% predicted) among both the COPDGene Phase-1 participants and 188 SPIROMICS Visit-1 participants, and validated the findings in an independent cohort of 363 COPDGene Phase-2 participants. Mediation analyses of multi-omic data examined the relationships between specific miR-126-3p and -p strands levels, their target mRNA and protein levels, and the severity of airflow obstruction.

**Results:**

Seventeen angiocentric proteins were increased in participants with PA/A > 1, with interleukin-1 receptor-like 1 (IL1RL1) and platelet-derived growth factor B (PDGFB) showing the most significant elevations. Among those with FEV₁ <50% predicted, eleven angiocentric proteins were increased, including IL1RL1, angiopoietin-2, and peroxiredoxin-5. Mediation analyses supported a contribution of reduced miR-126 levels to lower FEV₁ via select angiocentric molecules, including the direct miR-126 target selenoprotein T. Additionally, LINC01506 and CAPZA1 had a mediation effect on multiple clinical outcomes, including FEV₁, DLCO, and hematocrit.

**Conclusion:**

In addition to their role in pulmonary vascular remodeling, miR-126–regulated angiocentric proteins are also linked to airflow limitation, highlighting their potential as candidate biomarkers for COPD-associated pulmonary hypertension.

**Supplementary Information:**

The online version contains supplementary material available at 10.1186/s12931-026-03622-5.

## Background

The development of pulmonary hypertension (PH) is a major risk factor for morbidity and mortality in patients with chronic obstructive pulmonary disease (COPD) [1], a common disease primarily caused by cigarette smoking (CS). COPD-PH is characterized by pulmonary vascular remodeling, including loss of capillary beds, smooth muscle hypertrophy, and extracellular matrix degradation and reorganization [2–5], which result in increased mean pulmonary artery pressure. A lack of biomarkers for pulmonary vascular remodeling hinders its early diagnosis. Endothelial cell injury from and subsequent adaptation to CS [1, 6] is likely to play a central role in pulmonary vascular remodeling, while also contributing to the COPD pathogenesis via lung inflammation and loss of distal airspaces. We set out to identify circulating biomarkers of endothelial injury and repair implicated in vascular tone and remodeling in individuals suspected of COPD-PH, and to test their association with the severity of airflow limitation and emphysema.

Among endothelium-derived modulators of vascular tone and remodeling, which we term ‘angiocentric molecules,’ many are under transcriptional control of microRNA-126 (miR-126), an endothelial-enriched microRNA with broad vascular effects. A known suppressor of the vascular endothelial growth factor (VEGF) inhibitor sprouty-related EVH1 domain containing 1 (SPRED1), miR-126 enhances the survival and repair of endothelial cells in large vasculature beds, including pulmonary arteries [7, 8]. Conversely, in the lung microvasculature, miR-126 limits angiogenesis and promotes apoptosis through a different target repertoire including L-type amio acid transporter (LAT1) (an mTOR inhibitor) and a disintegrin and metalloprotease domain-9 (ADAM9) (a secreted enzyme that regulates cell fate) [9]. The divergent, cell-type-specific effects of miR-126 could in part be explained by the distinct sequences and targets (molecules directly inhibited) of its two strands, miR-126-3p and miR-126-5p [10]. We and others have identified that miR-126, which is directly suppressed by CS exposure [11], is decreased in COPD lungs [11], whereas its target ADAM9 is increased in COPD [12, 13] and in the lung perivascular areas of COPD-PH [12]. Furthermore, ADAM9 mediates CS-induced emphysema in mice [9, 14], a phenotype that shares with PH the loss of capillary beds. These findings suggest that miR-126-targets and molecules with similar angiocentric functions may be associated with pulmonary vascular remodeling and COPD-PH and play a role in the pathogenesis of COPD. Furthermore, the prevalence of COPD-PH is markedly higher (> 80%) in COPD individuals with severe airflow obstruction, defined as FEV_1_ <50% predicted (pp) measured post-bronchodilator via spirometry [15, 16], highlighting a potential pathogenic link.

A major challenge in diagnosing COPD-PH is that the gold standard - right heart catheterization - is invasive. In the absence of approved therapies for COPD-PH, there is reluctance to use this procedure, limiting both clinical evaluation and research efforts, and contributing to the scarcity of pulmonary vascular hemodynamic data in large cohorts of individuals with suspected COPD-PH. Even echocardiography, which is one of the most common and effective non-invasive screening methods for PH, has limited accuracy in determining the peak tricuspid regurgitation, which is used to estimate the mean right ventricular systolic pressure, when patients have concomitant COPD [17]. Thus, it is crucial to have additional non-invasive screening tools that clinicians can use to assess the pre-test probability of PH in patients with COPD.

Another complementary measurement indicative of COPD-PH is an increased ratio of pulmonary artery (PA) to the adjacent descending aorta diameter (PA/A) > 1 on chest computed tomography (CT) scans [18, 19], which is recommended as one of the screening tests for PH by the Pulmonary Vascular Research Institute guidelines [20]. As reviewed by Wells and Dransfield, the utility of PA/A ratio > 1 as a surrogate marker for COPD-PH relies on good inter- and intraobserver agreement and a strong and independent correlation with the mean PA pressure, especially in those with pulmonary disease, even after correction for sex, body surface area, and total lung capacity [21]. While the main PA diameter > 29 mm has a sensitivity, specificity, and positive predictive value of 84%, 75%, and 97%, respectively, for a mean pulmonary arterial pressure (mPAP) ≥ 25 mmHg [22], a PA/A > 1 has a sensitivity, specificity, and positive predictive value of 70%, 92%, and 96%, respectively, for a mPAP ≥ 20 mmHg [23], which is the new hemodynamic threshold for PH [24]. We therefore utilized PA/A >1 as the main surrogate marker of PH in this study.

Several circulating molecules, recently reviewed in [25], have been proposed as potential biomarkers of COPD-PH, including cardiac strain markers (e.g., NT-proBNP), inflammatory cytokines, nitric oxide, growth factor pathways (e.g., VEGF and FGF), and extracellular matrix–related proteins. However, these candidates have emerged from studies that have used heterogeneous definitions of COPD-PH, and even though they relied on echocardiography or small right-heart catheterization, they had limited sample sizes and lacked validation. Large multi-center COPD cohorts such as COPDGene and SPIROMICS provide an opportunity to address these limitations.

We hypothesized that the miR-126 targets and proteins with similar functions of modulating vascular tone and remodeling will be enriched in individuals with suspected COPD-PH, defined by PA/A > 1 as a surrogate marker for PH, and will be associated with airflow obstruction and emphysema. Using two large cohorts of individuals with a history of CS exposure, we conducted multi-modal analysis by integrating plasma proteomics (SomaScan 1.3 K and 5 K), transcriptomics, miRNA levels, CT imaging, and spirometry to first evaluate the relative abundance of angiocentric proteins in those with PA/A > 1, FEV_1_ <50 pp, and evidence of emphysema based on CT measurements. Then, we conducted a mediation analysis to investigate potential causal pathways linking the dysregulation of specific miR-126-3p and miR-126-5p strands to clinical outcomes. Our results show that targets of these strands are associated not only with suspected COPD-PH but also with the severity of airflow obstruction.

## Research design and methods

### Cohort descriptions

**Fig. 1:**
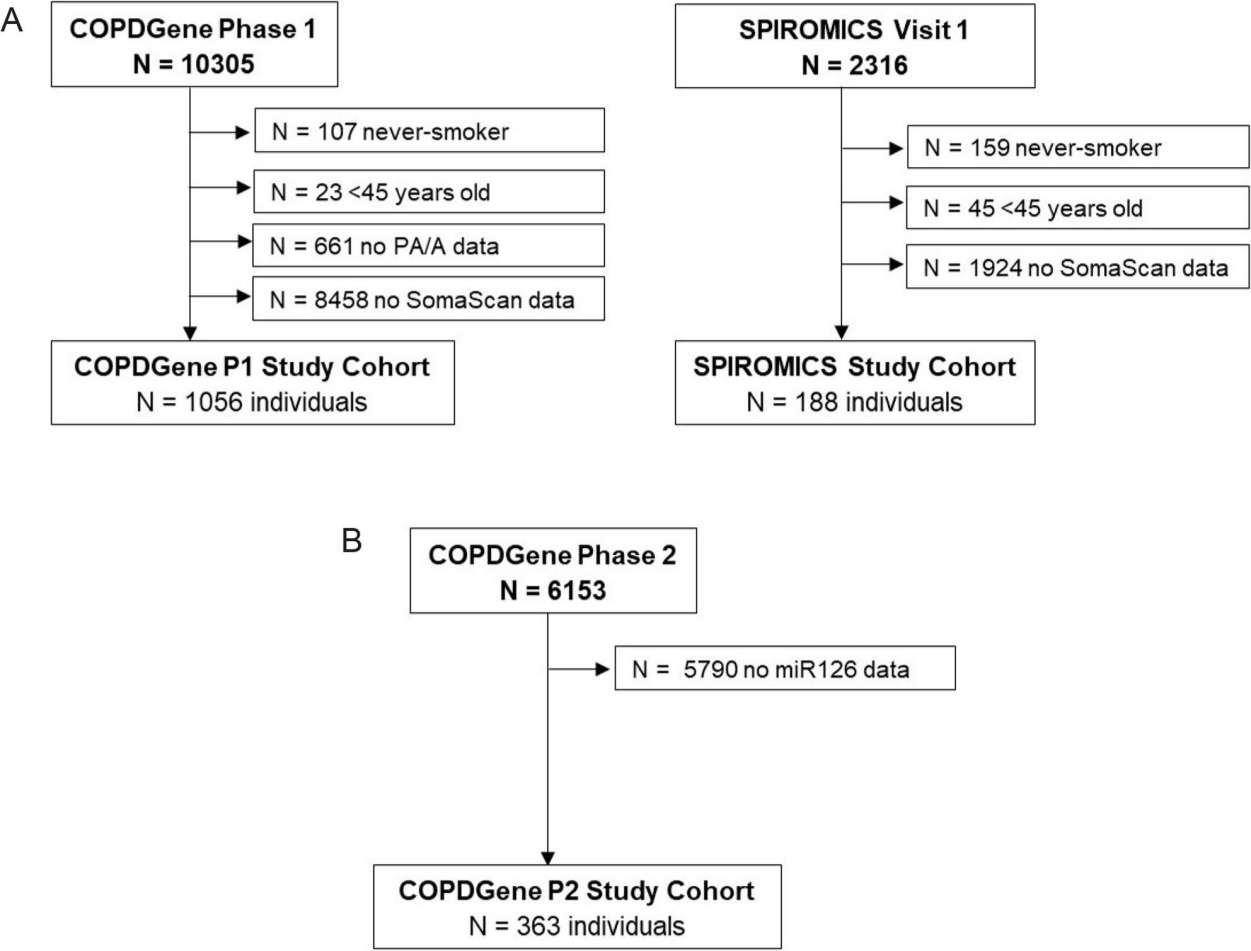
Diagram of Study Cohorts.** A**. Inclusion criteria for the angiocentric protein abundance study were individuals from COPDGene Phase 1 (P1) and SPIROMICS Visit 1 who were >45 years old, were ever-smokers, and had SomaScan 1.3K plasma proteomic data. Only COPDGene P1 had available PA/A data.** B**. Inclusion criteria for the mediation analysis were individuals from the COPDGene Phase 2 (P2) cohort who had miR126, proteomic, and transcriptomic data

COPDGene (ClinialTrials.gov Identifier: NCT00608764) is a multicenter study that enrolled 10,198 participants in Phase 1 between 2007 and 2011 who had a smoking history of 10 pack-years or greater, with or without COPD, and no respiratory exacerbations for > 30 days [26], as well as 107 never-smokers (smoked < 100 cigarettes). The cohort was recruited in a 2:1 non-Hispanic White: Black ratio. At baseline, participants who agreed and consented to participate in an omics ancillary study provided an additional sample of blood, which was collected using an 8.5 mL p100 tube (Becton Dickinson and Company). COPDGene Phase 2 was conducted between 2013 and 2017, as a five-year follow-up on 6153 subjects from Phase 1. The angiocentric protein abundance study included all (1056) individuals from COPDGene Phase 1 ever-smokers who were at least 45 years of age and had PA/A and proteomic SomaScan 1.3 K data **(**Fig. 1A**)**. The mediation analysis study included all (363) individuals from COPDGene Phase 2 who had miR-126 levels [27], SomaScan 5 K proteomic, and gene expression data [28] **(**Fig. 1B**)**.

The Subpopulations and Intermediate Outcome Measures in COPD study (SPIROMICS) (ClinialTrials.gov Identifier: NCT01969344) is a multicenter COPD study that enrolled 2788 participants of all races between 2010 and 2015 with a smoking history of 20 pack-years or greater, with or without COPD, and no respiratory exacerbations for > 30 days; and 200 never smokers (< 1 pack-year) [29]. At Visit 1, 2316 individuals provided fasting blood samples collected in a p100 tube and consented to the use their clinical and biomarker data. Of these, for the angiocentric protein abundance study, we included all (188) individuals who were ever smokers, were at least 45 years of age, and had SomaScan 1.3 K data **(**Fig. 1A**)**.

Both studies were approved and reviewed by the institutional review boards at all participating, coordinating, and reading centers and obtained signed consents from participants.

### COPD-PH phenotype definition

Given the absence of echocardiogram data (from the majority of the sites) or right heart catheterization data in the cohorts studied, we used PA/A > 1 as a radiographic indicator of PH [30–32]. PA/A ≤ 1 was defined as the absence of radiographic indicator of COPD-PH. In addition, the PA diameter was also evaluated as a continuous variable. Presence of severe airflow limitation was defined as post-bronchodilator FEV_1_ <50 pp (GOLD Stage 3 and 4), with the absence of severe airflow limitation defined as a post-bronchodilator FEV_1_ ≥50 pp (GOLD Stage 0–2). The presence of emphysema was defined as ≥ 5% of the lung volume occupied by CT values of low attenuation areas (the percent of lung tissue voxels) ≤ − 950 (LAA − 950) Hounsfield units (HU), while the absence of emphysema was defined as an LAA − 950 < 5% [33–35]. Low diffusion capacity of the lungs for carbon dioxide (DLCO), a potential indicator of both COPD-PH and emphysema phenotype in COPD, and high hematocrit, a potential indicator of COPD-PH [16], were also included as secondary outcomes in mediation analyses. Not all these phenotypic outcomes were available across all included data sets (Supplemental Table 1). Both COPDGene and SPIROMICS data sets had post-bronchodilator FEV_1_ pp data, whereas PA/A data were available only in COPDGene Phase 1 (Supplemental Table 1).

### Protein analysis

We included in our study 209 regulators of vascular endothelial function (which we refer to as angiocentric molecules) encompassing direct targets of miR-126, as well as functionally related molecules (Supplemental Table 2). MiR-126-targets included those predicted by TargetScan [36] as well as those previously experimentally confirmed in human lung microvascular endothelial cells [11]. Non-selected asangiocentric proteins were defined as the "Other" 1096 proteins in the SomaScan 1.3 K platform.

For COPDGene and SPIROMICS, levels of 1305 human proteins in the circulating plasma were measured using SomaScan Human Plasma 1.3 K assay (SomaLogic; Boulder, CO, USA), a multiplex aptamer-based assay [37]. Details on the 1.3 K data has been published [38]. COPDGene Phase 2 utilized SomaScan Human Plasma 5k assay, which is also a multiplex aptamer-based assay that measures 4979 human proteins. SomaScan data was standardized by SomaLogic per their protocol, consisting of plate hybridization, median signal normalization, and plate scaling, and was calibrated to a global reference [39–42].SomaScan version 4.0 (5k) has an additional normalization; median normalization to a reference using adaptive normalization by maximum likelihood is applied within dilution groups. Somamers were calibrated to control for variability across array signals, inter-run variability, inter-assay variation between analytes and batch differences between plates, and for SomaScan version 4 to quality control individual samples removing edge effects and technical variance. For analysis, protein levels were Log_2_ transformed.

### mRNA and miRNA analysis

mRNA data were obtained by RNA sequencing of peripheral whole blood from 3,618 COPDGene individuals in COPDGene Phase 2. Briefly, total RNA was extracted using the PAXgene blood miRNA kit, cDNA libraries were created using TruSeq Stranded Total RNA with Ribo-Zero Glboin Kit, and RNA-seq data underwent quality control as described [28]. Small RNA Libraries were constructed using NEXTFLEX Small RNA-Seq version 3 Automation Kit. To ensure comparability across samples, miRNA read counts were downsampled to match the sample with the lowest sequencing depth: 2.1 million total reads in the large cohort and 7.97 million in the pilot samples using the R package metaseqR (R Foundation for Statistical Computing, Indianapolis, IN). Technical replicates were collapsed with the DESeq2 Collapse Replicates function in R or by averaging the counts for each miRNA.

### Statistical analysis

Baseline characteristics were compared using Chi squared tests or Fisher’s exact tests for categorical data, a 2-sample t-test for normally distributed continuous variables, and Wilcoxon rank sums tests for non-normal continuous variable. Correlation between PA/A and FEV_1_ pp was calculated using Pearson correlation and results were presented as scatter plot. Cross-sectional analysis of differential angiocentric protein abundance and "other" protein abundance in the phenotypes PA/A > 1, FEV_1_ <50% pp, and LAA − 950 ≥ 5% was conducted using multivariable linear regression with target proteins as the outcome, controlling for age, sex, race, body mass index (BMI), and current CS status. The same multivariable model was used to assess the relationship of PA diameter with aniogentric protein abundance. A Benjamini-Hochberg adjusted p-value < 0.05 denoted FDR-significant results and a nominal p-value < 0.05 was used to define nominal significance. These analyses were conducted for the angiocentric and "other" proteins. The results of relative angiocentric protein abundance in those with FEV_1_ <50 pp and LAA − 950 ≥ 5% from COPDGene and SPIROMICS were combined into a meta-analysis that was conducted using an inverse variance weighted random-effect-model. Variance between studies was estimated using the DerSimonian–Laird estimator. A Benjamini-Hochberg adjusted FDR p-value < 0.05 was used to determine significance.

Using Metascape [43] (accessed on 1/13/26; version v3.5 20250701), an over-representation enrichment analysis was performed on proteins that were nominally significantly (*p* ≤ 0.05) associated with PA/A > 1, FEV_1_ <50 pp, and LAA − 950 ≥ 5% in COPDGene. Background proteins were the 209 angiocentric proteins and 1096 “other” proteins. The three result sets were input into Metascape to facilitate meta-analysis of overlapping pathways and enable direct comparison of pathway enrichment between the three groups. The enrichment analysis was set to a minimum overlap of 3, a p-value cut-off of 0.01, and a minimum enrichment of 1.5. Ontology sources used for the analysis were: GO Biological Processes, KEGG Pathway, GO Molecular Functions, GO Cellular Components, Reactome Gene Sets, Hallmark Gene Sets, Canonical Pathways, BioCarta Gene Sets, WikiPathways, and PANTHER Pathway. In addition, we performed protein-protein interaction enrichment analysis for physical core interactions. Briefly, if the resulting network contained at least three proteins and interactions with at least one other protein, the Molecular Complex Detector (MCODE) algorithm was applied to identify densely connected network component and enrichment analysis was applied to the component.

Two mediation models were fitted: (1) miR-126 →gene → outcome and (2) miR-126 → protein → outcome. The models were run for three distinct outcomes: FEV_1_ pp, DLCO, and hematocrit levels, and adjusted for covariates including age, sex, race, and smoking status. We employed the “lavaan” package [44] for mediation analysis rather than the traditional Baron and Kenny sequential approach [45] for two primary reasons: (1) “lavaan” directly tests the indirect effect and provides bootstrap confidence intervals, which offer greater statistical power and more accurate inference than sequential regression tests, particularly important given our limited sample size. (2) “lavaan” simultaneously estimate all model paths produces more precise standard error estimates than sequential regressions.

### Statistical software

Correlation and cross-sectional multivariable analysis were conducted in SAS (version 9.4, Stat 15.1). Meta-analyses were conducted in R (version 4.1.3) with Metafor (Version 3.8-1) package and individual meta-analyses forest plots were generated with Meta (version 6.1-0) package. Volcano plots were generated with Ggplot2 (version 3.4.3) packages and the baseline characteristic table using the TableOne (version 0.13.2) package.

## Results

### Study population included in analyses of angiocentric protein abundance in individuals with suspected COPD-PH or COPD with severe airflow limitation

The inclusion criteria for the individuals with indicators of suspected COPD-PH (PA/A > 1) or severe airflow limitation (FEV_1_ <50 pp) **(**Fig. 1A**)** were met in 1056 individuals from COPDGene Phase 1 and 188 individuals from SPIROMICS Visit 1. The baseline characteristics of these cohorts are described in Table 1. There were no significant differences between individuals in COPDGene and SPIROMICS in mean age (61.82 vs. 62.03 years, respectively; *p* = 0.767), sex (49.5% vs. 54.8% male; *p* = 0.211), body mass index (BMI) (28.71 vs. 28.06 kg/m^2^; *p* = 0.172), current smoking status (38.4% vs. 38%; *p* = 0.986), FEV_1_ to forced vital capacity ratio (FEV_1_/FVC) (0.65 vs. 0.64; *p* = 0.317), or the presence of emphysema as defined by LAA − 950 ≥ 5% on CT (30.1% vs. 27.7%; *p* = 0.56). The FEV_1_ pp was slightly lower in the COPDGene group (76% vs. 81%; *p* = 0.02). The majority of individuals in COPDGene and SPIROMICS were non-Hispanic white: 89.6% and 71.8%, respectively. COPD-PH presence based on PA/A > 1 was observed in 11.9% of COPDGene participants (not available in SPIROMICS); and 18% of COPDGene and 13.8% of SPIROMICS participants had an FEV_1_ <50 pp, with 30.1% of COPDGene and 27.5% of SPIROMICS having an LAA − 950 ≥ 5%. Details about the comparison between the cohorts of interest investigated in this study and the remainder of the COPDGene cohort are provided in Supplemental Table 3, with significant differences noted in age, sex, race, current smoking status, FEV1/FVC, and the rate of diabetes mellitus.

**Table 1:** Characteristics of Subjects in the Angiocentric Protein Abundance Study

	Study Cohort	COPDGene	SPIROMICS	
	Number of Subjects	1056	188	
	**Demographics**	**Mean (SD) or N (%)**	**Mean (SD) or N (%)**	**P-Value**
	Age (years)	61.82 (9.16)	62.03 (8.14)	0.77
	Males	523 (49.5%)	103 (54.8%)	0.21
Race	Non-Hispanic White	946 (89.6%)	135 (71.8%)	< 0.001*
	Black	110 (10.4%)	40 (21.3%)	
	Other	0 (0.0%)	13 (6.9%)	
	BMI	28.71 (6.13)	28.06 (4.93)	0.17
Smoking Exposure	Current Smoking	405 (38.4%)	71 (38.0%)	0.99
	ATS Pack-Years (median [IQR])	40.30 [27.98, 56.00]	40.00 [30.00, 53.50]	0.23**
Pulmonary Function and CT Evidence of Emphysema	FEV_1_% Predicted	76.47 (26.01)	81.25 (24.89)	0.020
	FEV_1_/FVC	0.65 (0.17)	0.64 (0.15)	0.32
	GOLD Stage			
	PRISm	103 (9.8%)	4(2.1%)	< 0.001
	0	448 (42.6%)	72 (38.3%)	
	1	94 (8.9%)	34 (18.1%)	
	2	215 (20.5%)	52 (27.7%)	
	3	125 (11.9%)	17 (9.0%)	
	4	66 (6.3%)	9(4.8%)	
	Percent Emphysema (LAA − 950) (median [IQR])	1.64 [0.46, 6.74]	2.50 [0.99, 6.03]	0.007
Study Groups	PA/A Ratio	0.84 (0.13)	N/A	
	PA/A Ratio > 1	126 (11.9%)	N/A	
	PA Diameter (cm)	2.69 (0.42)	N/A	
	FEV_1_ <50% predicted	192 (18.3%)	26 (13.8%)	0.17
	Presence of Emphysema (LAA − 950 ≥ 5%)	316 (30.1)	52 (27.7%)	0.56
Comorbidities	Congestive Heart Failure	35 (3.3%)	1 (0.5%)	0.032*
	Coronary Artery Disease	82 (7.8%)	6 (3.2%)	0.038
	Diabetes Mellitus	115 (10.9%)	17 (9.1%)	0.56
	Hypertension	462 (43.8%)	87 (46.8%)	0.49

Participant baseline characteristics were compared using Chi Square tests or Fisher Exact Test (*) for categorical data, 2-sample t-tests for normally distributed continuous variables, and Wilcoxon Rank Sums tests for non-normal continuous variables (**). N/A indicates not available

Number of observations missing a value for a variable: COPDGene: pulmonary function (*n* = 5), emphysema (*n* = 6). SPIROMICS: current smoker (*n* = 1), congestive heart failure (*n* = 2), coronary artery disease (*n* = 2), diabetes mellitus (*n* = 2), hypertension (*n* = 2)

### Angiocentric protein abundance in plasma of individuals with suspected COPD-PH

**Fig. 2:**
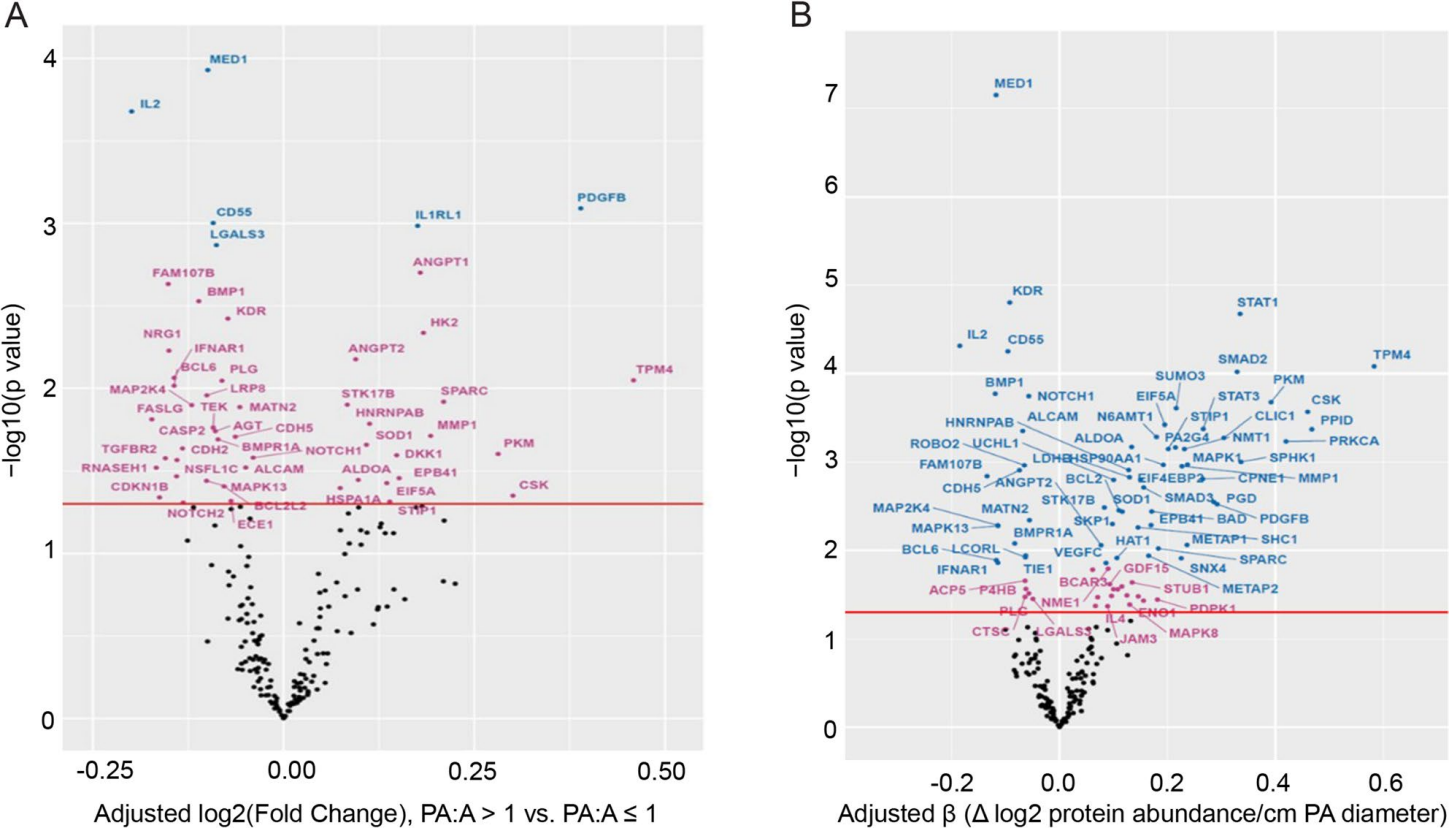
Relative Angiocentric Protein Abundance in Subjects with PA/A >1 and Correlation with PA size. **A**. Volcano plot of differential protein abundance enriched in those with PA/A >1 vs. PA/A ≤1, using multivariable linear regression with angiocentric proteins as outcome and PA/A >1 as variable. Covariates included age, sex, race, body mass index, and current smoking status. **B**. Volcano plot of beta estimate levels of angiocentric protein abundance (log 2-fold change) with the PA diameter (as independent continuous variable) using linear regression controlled for age, sex, race, body mass index, and current smoking status. A Benjamini-Hochberg-adjusted p-value <0.05 was used to define results that were FDR-significant (blue), a p-value <0.05 was used to define results that were nominal significant (pink) or non-significant (NS, black)

Because miR-126 levels are decreased in the lungs of patients with COPD [12], we hypothesized that miR-126-target proteins and functionally similar proteins involved in pulmonary vascular remodeling, which we collectively termed “angiocentric” proteins, are enriched in our patient cohort and are increased in abundance in those with PA/A > 1. A differential protein abundance analysis conducted using PA/A > 1 as a predictor identified six target proteins that had significantly changed levels (FDR < 0.05) in those with PA/A > 1. Of these, interleukin-1 receptor-like 1 (IL1RL1) and platelet-derived growth factor subunit B (PDGFB) were increased in abundance, while mediator of RNA polymerase II transcription subunit 1 (MED1), interleukin-2 (IL2), complement decay-accelerating factor (CD55), and galectin-3 (LGALS3) were decreased in those with PA/A > 1 (Fig. 2A, Supplemental Table 4). There were 17 additional target proteins which were nominally (*p* < 0.05, FDR > 0.05) increased in those with PA/A > 1: angiopoietin-1 (ANGPT1), hexokinase-2 (HK2), tropomyosin 4 (TPM4), angiopoietin-2 (ANGPT2), osteonectin/secreted protein acidic and cysteine rich (SPARC), serine/threonine-protein kinase (STK17B), heterogenous nuclear ribonucleoprotein A/B (HNRNPAB), matrix metallopeptidase 1 (MMP1), superoxide dismutase (SOD1), pyruvate kinase M (PKM), Dickkopf signaling pathway inhibitor 1 (DKK1), erythrocyte membrane protein band 4.1 (EPB41), fructose-bisphosphonate aldolase A (ALDOA), eukaryotic translation initiation factor 5 A-1 (EIF5A), heat shock 70 kDa protein 1 A (HSPA1A), tyrosine-protein kinase (CSK), and stress-induced phosphoprotein 1 (STIP1) (Fig. 2A, Supplemental Table 4).

In a separate analysis, we used the PA diameter as a continuous variable, instead of a strict PA/A > 1 criterion (Fig. 2B), with the full list of significantly (FDR < 0.05) and nominally (*p* < 0.05, FDR > 0.05) associated angiocentric proteins provided in Supplemental Table 5. Fifty-five target proteins and 143 non-target proteins were significantly associated with both PA/A ratio and with the PA diameter, with the beta estimate being significantly higher for the PA/A measurement (*p* = 1.9E-07). Of these overlapping proteins demonstrating moderate or greater positive associations with PA included TPM4, peptidyl-prolyl cis-trans isomerase D (PPID), CSK, protein kinase C alpha type (PKC-A), PKM, sphingosine kinase 1 (SPHK1), signal transducer and activator of transcription 1 (STAT1), SMAD family member 2 (SMAD2), chloride intracellular channel protein 1 (CLIC1), PDGFB, PDG, STAT3, copine-1 (CPNE1), mitogen-activated protein kinase 1 (MAPK1), glycylpeptide N-myristoyltransferase (NMT1), MMP1, small ubiquitin-related modifier 3 (SUMO3), and STIP1 (Supplemental Tables 4, 5). Interleukin-2 (IL-2), protein FAM107B (FAM107B), bone morphogenetic protein 1 (BMP-1), MED1, B-cell lymphoma 6 protein (BCL6), dual specificity mitogen-activated protein kinase kinase 4 (MP2K4), interferon alpha/beta receptor 1 (IFN-a/b R1), and mitogen-activated protein kinase 13 (MK13), followed by cadherin-5 (CDH5), bone morphogenetic protein receptor type-1 A (BMPR1A), vascular endothelial growth factor receptor 2 (VEGFR2), and complement decay-accelerating factor (DAF) showed significant, albit weaker inverse association with PA diameter. Reactome Pathway enrichment analysis revealed a strong convergence on growth factor and kinase-driven signaling networks, with the most significant pathways centered on PI3K/AKT and MAPK/RAF signaling, including constitutive PI3K activation, RAF/MAPK cascades, MAPK1/3 signaling, and multiple RAS, RAF, and BRAF mutant pathways, indicating robust activation of proliferative and stress-response signaling axes. These were complemented by enrichment in pathways related to immune and inflammatory responses, particularly neutrophil and platelet degranulation and IL-4/IL-13 signaling, as well as receptor-mediated processes such as AGE–RAGE and KIT signaling. Additional enrichment was noted in cell migration and interaction pathways, including EPH-ephrin signaling, and extracellular matrix remodeling.

### Angiocentric protein abundance in plasma of individuals with severe airflow limitation and emphysema

**Fig. 3:**
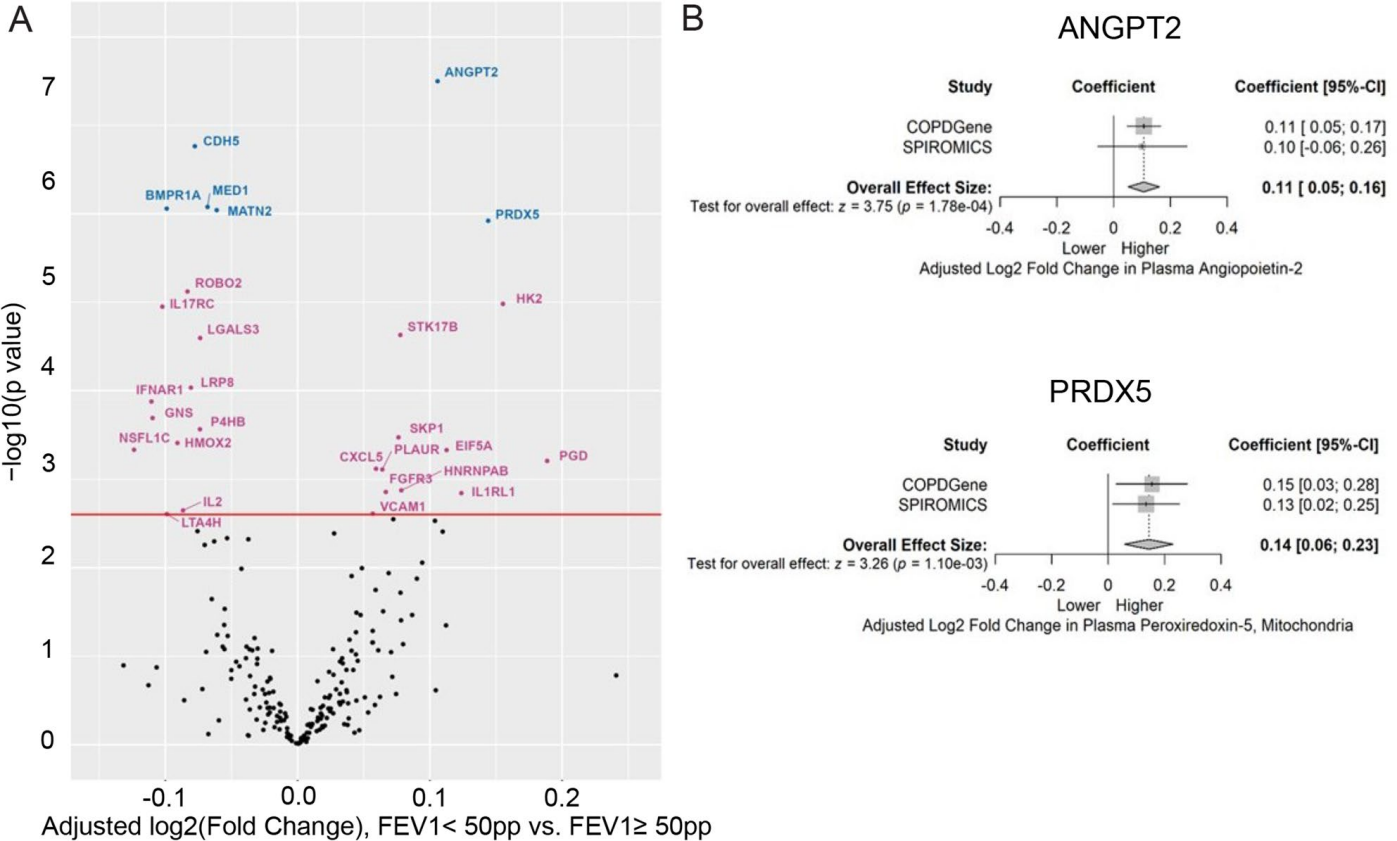
Relative Angiocentric Protein Abundance in Subjects with FEV_1_ <50 pp. **A**. Volcano plot of differential protein abundance (log 2-fold change) in those with FEV_1_ <50 pp vs. those with FEV_1_ 50 pp using meta-analysis of data from COPDGene and SPIROMICS. A Benjamini-Hochberg-adjusted p-value <0.05 was used to define results that were FDR-significant (blue), a p-value <0.05 was used to define results that were nominal significant (pink) or non-significant (NS, black).** B**. Forest plots of angiocentric proteins that were significantly (FDR<0.05) more abundant in those with FEV1 <50 pp compared to those with FEV1 ≥50 pp in COPDGene, SPIROMICS, and in the meta-analysis, conducted using an inverse variance-weighted random-effects model. Variance between studies was estimated using the DerSimonian-Laird estimator

We hypothesized that angiocentric proteins implicated in pulmonary vascular remodeling are also increased in individuals with FEV_1_ <50 pp. Combining the data available from both COPDGene Phase 1 and SPIROMICS Visit 1, we identified six proteins which were significantly associated (FDR < 0.05) with FEV_1_ <50 pp: angiopoietin-2 (ANGPT2) and mitochondrial peroxiredoxin-5 (PRDX5) were increased, while CDH5, MED1, matrilin-2 (MATN2), and BMPR1A were decreased in individuals with FEV_1_<50 pp (Fig. 3A, Supplemental Table 6). The coefficient of variation in protein abundance was consistent across individuals from COPDGene and SPIROMICS for all 6 proteins (Fig. 3B). Additionally, there were 11 angiocentric proteins nominally (*p* < 0.05; FDR > 0.05) increased, and 11 angiocentric proteins nominally decreased in individuals with FEV_1_ <50 pp (Fig. 3A, Supplemental Table 6).

Six angiocentric proteins were increased in individuals with both PA/A > 1 and FEV_1_ <50 pp. HK2, STK17B, eukaryote translation initiation factor 5 A-1 (EIF5A), and HNRNPAB were nominally increased in individuals with both phenotypes. ANGPT2 was nominally increased in individuals with PA/A > 1 and FDR-significantly increased in FEV_1_ <50 pp. IL1RL1 was FDR-significantly increased in individuals with PA/A > 1 and nominally increased in FEV_1_ <50 pp.

Five angiocentric proteins were decreased in individuals with both PA/A > 1 and FEV_1_ <50 pp: MED1 was FDR-significantly associated with both phenotypes; BMPR1, MATN2, and CDH5 were nominally associated with PA/A > 1 and FDR-significantly associated with FEV_1_ <50 pp; low-density lipoprotein receptor-related protein 8 (LRP8) was nominally associated with both.

A separate differential protein abundance analysis for the other proteins not included in the angiocentric set was conducted for PA/A > 1 and FEV_1_ <50 pp (Supplemental Fig. 1A, 1B). There were 11 proteins that were significantly increased (FDR < 0.05) in individuals with both PA/A > 1 and FEV_1_ <50 pp: cardiac type troponin T2 (TNNT2), complement 7 (C7), serpin family A member 3 (KLK3/SERPINA3), oxidized low density lipoprotein receptor 1 (OLR1), matrix metallopeptidase 9 (MMP9), thrombospondin 2 (THBS2), neutrophil expressed elastase (ELANE), S100 calcium binding protein A9 (S100A9), C-X-C motif chemokine ligand 12 (CXCL12), bactericidal permeability increasing protein (BPI), and angiopoietin-like 4 (ANGPTL4) (Supplemental Fig. 1A, 1B).

**Fig. 4:**
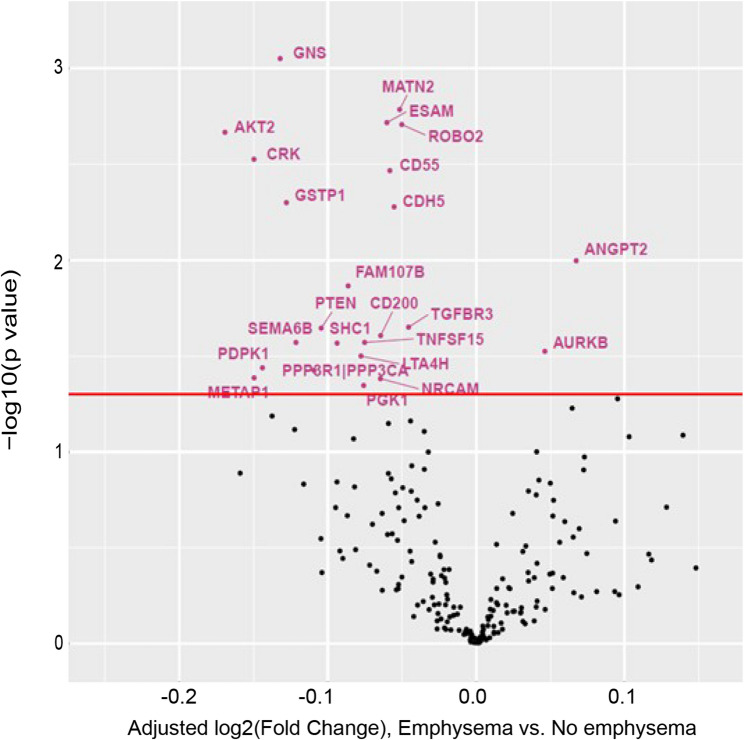
Relative Angiocentric Protein Abundance in Subjects with Emphysema. Volcano plot of differential protein abundance (log 2-fold change) in individuals with emphysema, defined as LAA -950 ≥5% vs those without emphysema, using meta-analysis of data from COPDGene and SPIROMICS; this was conducted using an inverse-variance-weighted random-effects model. A Benjamini-Hochberg-adjusted p-value <0.05 was used to define results that were FDR-significant; a p-value <0.05 was used to define results that were nominal significant (pink) or non-significant (black)

We then assessed emphysema, defined as an LAA − 950 ≥ 5% on CT, as another phenotype of severe COPD, complementary to aiflow limitation (FEV_1_ <50 pp). Individuals from COPDGene Phase 1 and SPIROMICS Visit 1 were included in this analysis. Although there were no FDR-significant differences in angiocentric protein abundance, ANGPT2 (which was also increased in individuals with PA/A > 1 and FEV_1_ <50 pp) and aurora kinase B (AURKB) were nominally (*p* < 0.05, FDR > 0.05) increased in abundance in individuals with emphysema compared to those without (Fig. 4, Supplemental Table 7). Of the 22 angiocentric proteins nominally decreased in individuals with emphysema, MATN2 and CDH5 were also decreased in individuals with PA/A > 1 and FEV_1_ <50 pp (Fig. 4, Supplemental Table 7).

### Biologic pathway enrichment analysis of angiocentric proteins in individuals with suspected COPD-PH, severe airflow limitation, and emphysema

**Fig. 5:**
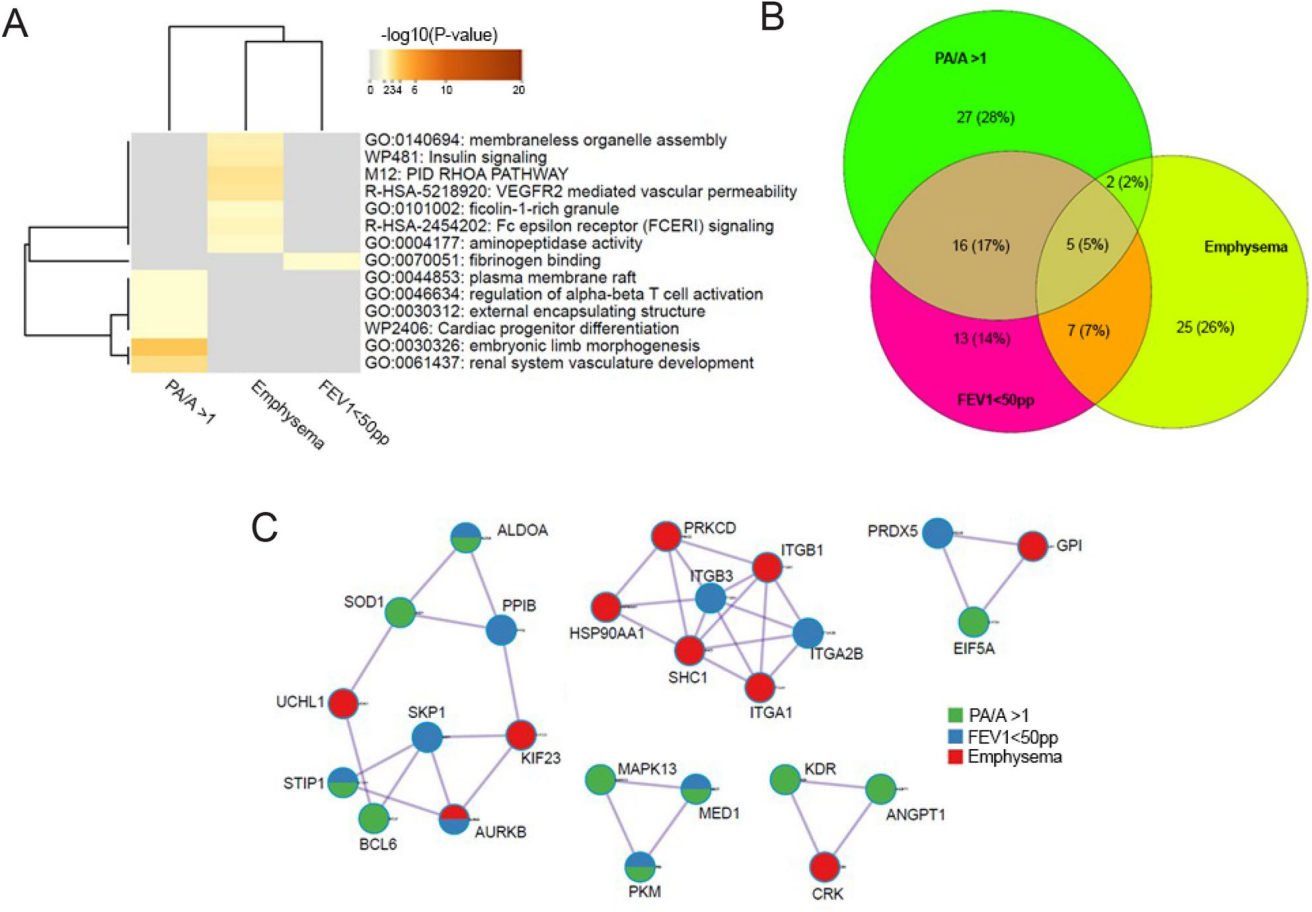
Pathway Enrichment Analysis of Angiocentric Proteins.**A**. Hierarchical clustering dendrogram showing the significantly enriched ontology clusters or pathways for the three clinical phenotypes: suspected COPD-PH defined as PA/A >1, severe airflow limitation defined as FEV_1_<50 pp, and emphysema defined as LAA -950 ≥5%. Heatmap cells are colored by their p-values, with gray cells indicating the lack of enrichment for that term in the corresponding gene/protein list. **B**. Euler diagram illustrating the number of angiocentric proteins that overlap with each category. All are nominally significant. **C**. Protein-protein interaction (PPI) networks highlighting interacting proteins from the input lists obtained by PPI enrichment analysis (using Metascape). Green: angiocentric proteins associated with PA/A >1. Blue: angiocentric proteins associated with FEV_1_<50 pp. Red: angiocentric proteins associated with emphysema

We performed an enrichment analysis to identify biologic pathways that involved the angiocentric proteins enriched in individuals with suspected COPD-PH (COPD with PA/A > 1), severe airflow limitation (FEV_1_ <50 pp), and emphysema (LAA − 950 ≥ 5%). Six pathway clusters were associated with PA/A > 1: renal system vasculature development (involving *n* = 6 angiocentric proteins), embryonic limb morphogenesis (*n* = 6), cardiac progenitor differentiation (*n* = 5), plasma membrane rafts (*n* = 5), regulation of alpha-beta T cell activation (*n* = 5), and external encapsulating structures (*n* = 10) **(**Fig. 5A**)**. The fibrinogen binding pathway was associated with FEV_1_ <50 pp, involving 3 angiocentric proteins **(**Fig. 5A**)**. Seven pathway clusters were associated with LAA − 950 ≥ 5%: Ras homolog family member A (RhoA) pathway (involving *n* = 5 angiocentric proteins), VEGFR2 mediated vascular permeability (*n* = 4), insulin signaling (*n* = 8), membraneless organelle assembly (*n* = 5), Fc episilon receptor signaling (*n* = 7), ficolin-1-rich granule (*n* = 8), and aminopeptidase activity (*n* = 3) **(**Fig. 5A**)**.

Next, we determined how many angiocentric proteins overlapped with these clinical phenotypes based on their functions or shared pathways **(**Fig. 5B**)**. Individuals with PA/A > 1 shared 16 angiocentric proteins with individuals with FEV_1_ <50 pp and 2 angiocentric proteins with individuals with LAA − 950 ≥ 5% **(**Fig. 5B, Supplemental Table 8). Five angiocentric proteins were common between all three phenotypes: CD55, ANGPT2, CDH5, AGT, and MATN2 (Fig. 5B, Supplemental Table 8). These results were all nominally significant (*p* < 0.05, FDR > 0.05).

Finally, a protein-protein interaction enrichment analysis revealed five networks of interconnected angiocentric proteins associated with PA/A > 1, FEV_1_ <50 pp, and LAA − 950 ≥ 5% **(**Fig. 5C**)**. Two networks included angiocentric proteins enriched in all three phenotypes: the first cluster involved PRDX5, EIF5A, and glucose-6-phosphate isomerase (GPI), and the second cluster involved ALDOA, SOD1, s-phase kinase-associated protein 1 (SKP1), peptidylprolyl isomerase B (PPIB), ubiquitin C-terminal hydrolase L1 (UCHL1), STIP1, BCL6, AURKB, and kinesin family member 23 (KIF23) **(**Fig. 5C**)**.

### Correlation between PA/A and FEV_1_

**Fig. 6:**
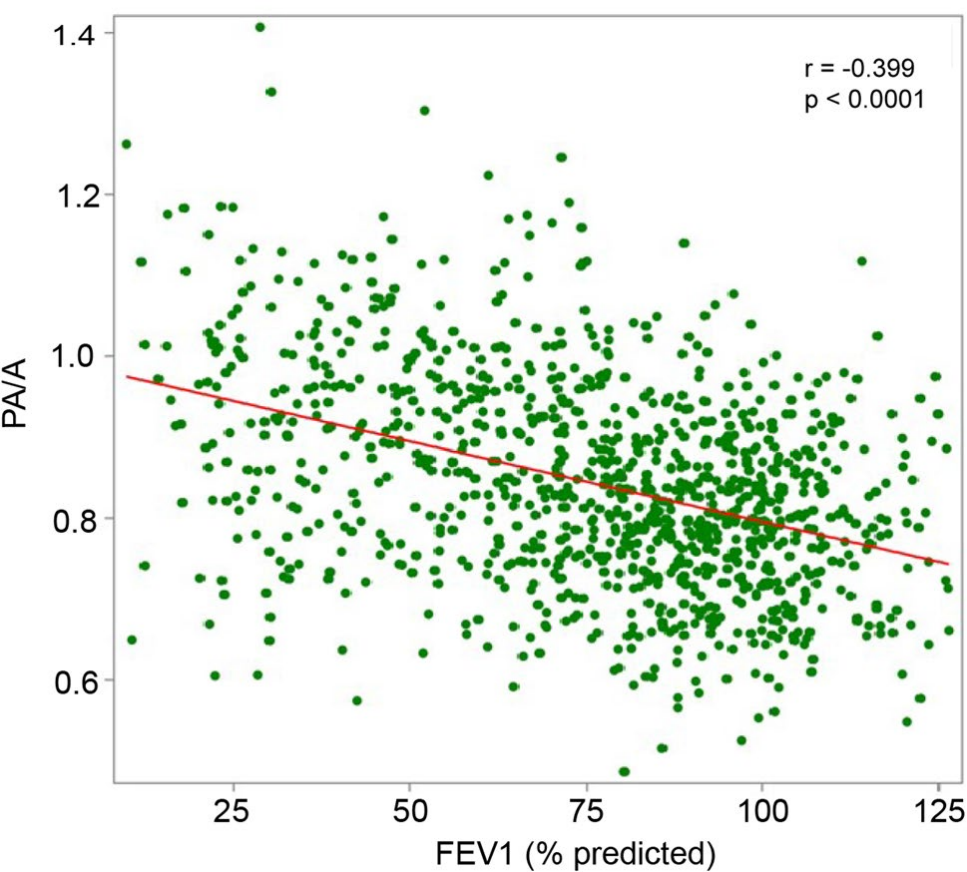
Correlation between FEV_1_ pp and PA/A. Pearson correlation dot plot and linear regression between FEV_1_ pp and PA/A ratio in the COPDGene Phase 1 dataset (*n* = 1056). Each dot represents an individual

Given the increased abundance of certain angiocentric proteins in individuals with both suspected COPD-PH (PA/A > 1) and severe airflow obstruction, we tested the relationship between PA/A and FEV_1_ pp in our cohort, using Pearson correlation analysis. We hypothesized that a higher PA/A is associated with more airflow limitation (i.e., lower FEV_1_ pp). For the 1056 individuals from COPDGene Phase 1 with both PA/A and FEV_1_ data, there was a statistically significant inverse correlation between FEV_1_ pp and PA/A (*r*= -0.399; *p* < 0.001) **(**Fig. 6**)**.

### Mediation analysis

To further validate the link between angiocentric proteins, many of which are miR-126-targets, and airflow obstruction or other COPD phenotypes, we next performed a mediation analysis of a cohort selected for the availability of miR126-, proteomic-, transcriptomic-, and phenotypic data. Demographic data for the 363 subjects from Phase 2 of the COPDGene cohort who met the inclusion criteria for the mediation analysis are presented in Supplemental Table 9. The subjects’ average age was 59.1 years, with most being female (54.5%; *n* = 198), non-Hispanic white (74.4%; *n* = 270), and having a mean BMI of 29.1 kg/m². The group included more former smokers (62.8%; *n* = 228) than current smokers (37.2%; *n* = 135). Analyses were conducted using FEV_1_, DLCO, and hematocrit as outcomes.

**Fig. 7:**
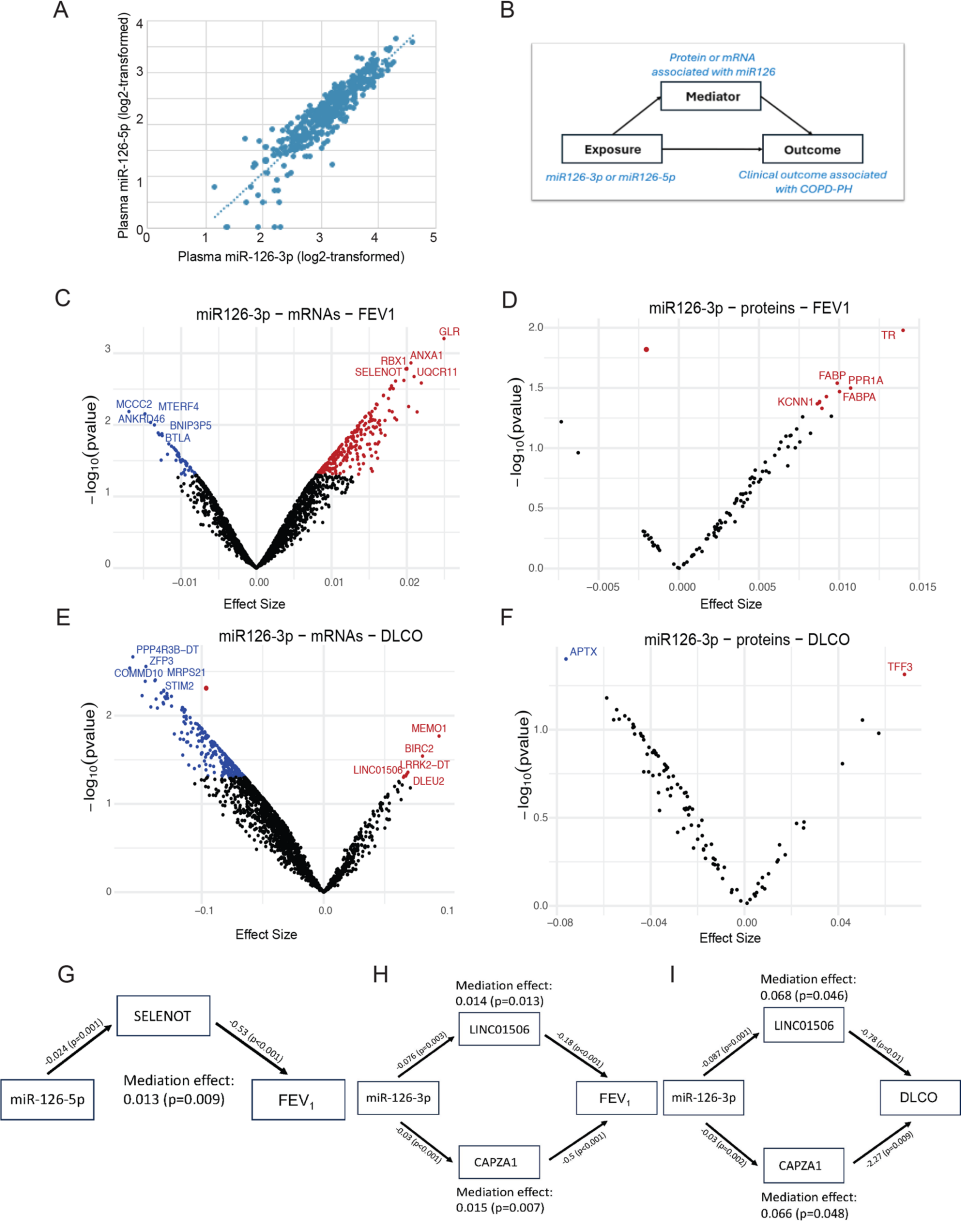
Evidence of Mediation of miR-126-Targets on COPD Phenotypes. **A**. Correlation between miR-126-3p and -5p levels (log 2-fold change).**B**. Schematic of the mediation analysis conducted on genes and proteins correlated with miR-126-3p and -5p, assessing the mediation effect on FEV_1_, DLCO, and hematocrit. **C-D**. Volcano plot showing the mediation effect on FEV_1_of genes **(C)** and proteins **(D)** associated with miR-126-3p. **E**. Mediation effect on FEV_1_ of the direct miR-126 target, SELENOT. **F-G**. Volcano plot showing the mediation effect on DLCO of genes **(F)** and proteins **(G)** associated with miR-126-3p. **H-I**. Indicated molecules associated with miR-126-3p were nominally significant mediators of both decreased FEV_1_and DLCO. In volcano plots, red denotes nominal significance (p-value <0.05); black denotes nonsignificant data (NS)

We first noted that the circulating levels of the two miR-126 strands, miR-126-3p and miR-126-5p, were highly correlated across all subjects **(**Fig. 7A**)**. We conducted two mediation models: miR-126 to gene to outcome and miR-126 to protein to outcome **(**Fig. 7A**)**. Since the direct effects of miRNA strands (-3p or -5p) on the transcriptome and the respective proteome are primarily inhibitory, we primarily focused on genes or proteins that were significantly negatively correlated with miR-126 levels. There were 1748 gene transcripts and 98 proteins that were significantly negatively associated with miR-126-3p levels, and 728 gene transcripts and 14 proteins that were significantly negatively correlated with miR-126-5p levels.

Although not reaching FDR significance in this relatively smaller and heterogeneous cohort, the mediation analyses identified targets that were nominally statistically significant (*p* < 0.05) in mediating the effect of the respective miR-126 strands on FEV_1_. Specifically, 229 genes and 8 proteins negatively correlated with miR-126-3p, and 99 genes and 3 proteins negatively correlated with miR-126-5p showed nominal significant mediation for decreased FEV_1_ (*p* < 0.05) **(**Fig. 7C and D**)**. Of these, 97 genes (98%) and all 3 proteins were significant mediators for both strands. SELENOT, encoding selenoprotein T and a known direct target of miR-126-5p, emerged as a nominally significant mediator for both miR-126 strands **(**Fig. 7C and G**)**. In the mediation pathway (Fig. 7E), lower miR-126 levels (modeled as the exposure) were associated with significantly higher SELENOT levels β = -0.02; *p* = 0.001, while higher SELENOT levels were associated with lower FEV_1_ values (β = −0.53; *p* < 0.001). The indirect (mediation) effect of miR-126 on FEV_1_ through SELENOT was statistically significant (β = 0.013, *p* = 0.009), calculated as the product of the miR-126→SELENOT and SELENOT→FEV_1_ associations. These findings indicate that SELENOT mediates the detrimental effect of low miR-126 levels on lung function: lower miR-126 levels are associated with increased SELENOT expression, which, in turn, is associated with reduced FEV_1_.

Using DLCO as the outcome of miR-126-targets, 7 genes and 2 proteins negatively associated with miR-126-3p, along with 3 genes negatively associated with miR-126-5p, were significant mediators for low DLCO, the latter being common for both strands **(**Fig. 7E and F**)**. Of the 448 genes and 5 proteins negatively correlated with miR-126-3p, 233 genes and 1 protein negatively correlated with both strands mediated a higher hematocrit, with 37 genes reaching an FDR < 0.05 level of significance (Supplemental Fig. 2A, 2B). Two genes that negatively correlated with miR-126-3p, the long intergenic non-protein coding RNA 1506 (LINC01506) and capping actin protein of muscle Z-line subunit alpha 1 (CAPZA1), were nominally significant mediators of all three outcomes tested: reduced FEV_1_ pp, reduced DLCO, and elevated hematocrit. (Fig. 7H-I, Supplemental Fig. 2C). Of note, the majority of subjects in the study were not polycythemic (Supplemental Fig. 2D).

## Discussion

In this study, we utilized large-scale, well-characterized COPD cohorts and integrated multi-omic approaches to examine the relative abundance of angiocentric proteins, mainly targets endothelial-enriched miR-126 in individuals with suspected COPD-PH. Our findings highlight a potential mechanistic link between endothelial dysfunction and features of pulmonary vascular remodeling in COPD. By demonstrating that both mature strands of miR-126 (miR-126-3p and miR-126-5p) are reduced in COPD, this study provides the clinical context for previous work showing that their transcriptional and proteomic targets are enriched in pathways related to angiogenesis, cell survival, and responses to oxidative stress [11]. By combining cross-sectional, correlation, and mediation analyses, we show that miR-126-associated molecular signatures are not only significantly enriched in individuals with suspected COPD-PH but also linked to the severity of airflow obstruction and decreases in pulmonary gas transfer in COPD. The inverse correlation between the PA/A ratio and FEV₁ in our cohort aligns with previous reports of increased PH prevalence in severe COPD [46, 47, 48, 16], support the biological plausibility of our findings.

Unlike pulmonary arterial hypertension (PAH), which has seen notable advances in understanding and treatment [49, 50], there remain significant gaps in knowledge regarding the pathogenesis of COPD-PH. Previous studies indicate that different PH types exhibit distinct metabolomic profiles [51], and COPD-PH has unique pathophysiological features. This underscores the importance of identifying biomarkers specific to COPD-PH. Our research fills this gap by examining a cohort with a history of significant cigarette smoke exposure who are at risk for or diagnosed with COPD. Given the considerable injury to endothelial cells and their maladaptive reactions to cigarette smoke exposure in COPD [1, 6], the endothelium is likely to play a key role in pulmonary vascular remodeling in COPD-PH. Although previous studies have implicated endothelial dysfunction in PH [52–55], few have directly linked specific endothelial-derived molecules, or an endothelial-enriched posttranscriptional regulatory network such as the miR-126 targetome, to clinical outcomes in large human cohorts. Our study stands out by leveraging two independent, well-characterized cohorts (COPDGene and SPIROMICS) with large discovery-phase sample sizes and cross-cohort replication.

Most conventional treatments for PAH exacerbate hypoxemia in COPD-PH since they vasodilate poorly ventilated lung units, thus worsening the ventilation-perfusion mismatch [56, 57]. Although lacking the occlusive plexiform lesions composed of endothelial and vascular smooth muscle cells found in PAH [58, 59], similar to PAH, the pulmonary arteries in COPD-PH undergo hypertrophy of both the intima and the tunica media [60]. Therefore, the angiocentric proteins most elevated in suspected COPD-PH, such as ANGPT2, HK2, and IL1RL1, which are also implicated in vascular proliferation, inflammation, and oxidative stress in PAH, suggest the presence of shared molecular drivers across these pulmonary vascular diseases.

The consistency of our findings regarding the enrichment of angiocentric proteins in individuals with severe airflow obstruction (FEV_1_ <50 pp) across the COPDGene and SPIROMICS cohorts supports a potential dual role in COPD-PH and COPD pathogenesis. Four angiocentric proteins were significantly associated with either PA/A > 1 or severe airflow limitation (FEV₁ <50 pp), IL1RL1, PDGFB, ANGPT2, and PRDX5. Given its important role in tissue remodeling, chronic inflammation has long been considered a key contributor to pulmonary vascular remodeling in COPD-PH, and elevations in circulating inflammatory cytokines have been reported in small cohorts of COPD-PH [61, 62]. The signaling of IL1RL1 (also known as ST2) and its ligand IL-33 has been mechanistically implicated in experimental PH [63, 64], providing high biological plausibility to our findings. Supporting human relevance, in a small cohort of almost ~ 90 individuals with COPD, serum IL-33 was measured at higher levels in echocardiography-defined COPD-PH [65], while soluble ST2 was higher during acute exacerbations in those with echocardiography-defined COPD-PH within a cohort of ~ 200 individuals with COPD [66]. The PDGF pathway’s role in pulmonary vascular remodeling has also been mechanistically established in animal models [67] and circulating PDGFB are elevated in human PH [68]. Besides promoting inflammation and tissue remodeling [69–71], ANGPT2 destabilizes endothelial junctions and, by engaging angiogenic sprouting [72], it may contribute to capillary dropout and rarefaction seen in both PH and emphysema. In a small cohort (~ 90 individuals), ANGPT2 was significantly elevated in idiopathic PAH [73] and a study of ~ 100 subjects with COPD found that ANGPT2 may have predictive value for echocardiography-defined COPD-PH, especially when combined with NT-proBNP [74]. Other targets of interest were PRDX5, an inducible antioxidant enzyme in mitochondria of pulmonary artery smooth muscle cells, which has been shown to attenuate hypoxia-induced oxidative stress and HIF signaling [75], and HK2, which plays a role in glycolysis and is increased during hypoxia-induced pulmonary vascular remodeling [76, 77]., while SELENOT, a redox-sensitive protein with antioxidant properties, may be critical for endothelial survival under oxidative stress [78].

Despite the strengths of our study, several limitations must be acknowledged. First, we relied on a non-invasive measure of PH presence (PA/A > 1) because of the lack of invasive hemodynamic and non-invasive 2D-echocardiographic data. Because right heart catheterization is not feasible in large clinical cohorts, using a PA/A > 1 as a surrogate marker for suspected COPD-PH is pragmatic and consistent with current guideline recommendations [79]. This approach enables biomarker discovery at a scale that would otherwise be impractical. Given the high specificity and positive predictive value of PA/A > 1 for PH, it is likely that most individuals classified in this group had underlying PH. However, the relatively low negative predictive value (52%) [23], indicates that the absence of PA/A > 1 does not exclude COPD-PH, raising the possibility that some individuals in the comparison group may also have had PH. Additionally, the PA/A ratio does not differentiate between types of PH, including those due to left heart dysfunction, which were present in 3.5% and 0.5% of individuals in the COPDGene and SPIROMICS cohorts, respectively, and could have influenced our results. Also, 43.8% and 46%, respectively, had HTN, which can increase arterial diameter and the PA/A ratio. This has led to a decreased clinical utility of the PA/A ratio in the general population, although its utility in COPD remains supported by multiple studies cited in our report. By conducting a separate analysis of proteomic association with the PA diameter, we partially mitigated this caveat. However, future studies incorporating hemodynamic validation in a subset of participants and conducting sensitivity analyses will help address this limitation.

Although our main goal was to identify potential circulating biomarkers of COPD-PH, any extrapolation of our findings to the pathobiology of pulmonary vascular remodeling should be approached cautiously. Our results agree with previous reports showing that circulating miR-126 and its targets track with tissue levels in smoking-related disease, being decreased by smoking in lung tissue and in lung microvascular endothelial cells, as well as in COPD [12, 80]. However, peripheral blood-derived data may not fully capture the complexity of the pulmonary vascular microenvironments, even with high-resolution strand-specific analyses. Notably, cigarette smoking exposure increases the release of circulating exosomes, which we have shown to be enriched in miR-126 [81]. Circulating miR-126 is also elevated during acute exacerbations of COPD [82], indicating that miR126 dynamics may reflect not only pulmonary vascular remodeling, but also right ventricular strain or systemic stress, inflammation, or vasculopathy. Future studies incorporating tissue-specific analyses, including exosome profiling from lung compartments, will be critical for validating and refining these circulating signatures.

To address the limitation that the cross-sectional design precludes temporal inference, we applied mediation modeling to explore plausible mechanistic pathways linking miR-126, its downstream targets, and clinical outcomes. However, we acknowledge that even with mediation analysis, causal relationships cannot be definitively established. This is a fundamental limitation of observational cohort studies—particularly in heterogeneous syndromes such as COPD, where disease mechanisms and phenotypes vary widely. Moreover, several of our findings were only nominally statistically significant, likely reflecting both biological heterogeneity and the limited number of subjects meeting inclusion criteria for specific analyses. We also acknowledge the impact of misclassification based on our inclusion criteria; because any misclassification is likely non-differential with respect to protein levels, its primary effect would be attenuation of true associations (bias toward the null) and reduced power to detect biologically meaningful signals, suggesting that our findings may underestimate the strength of true proteomic relationships. Together, these limitations along with the demographic characteristics of the cohorts analysed, underscore important future directions, including prospective validation in well-sub-phenotyped cohorts with complete data sets that incorporate right heart catheterization, echocardiography, well-defined gas exchange abnormalities, lung tissue-specific expression profiling, and longitudinal sampling. Such studies, conducted across diverse populations, will be critical for validating these biomarkers and assessing their utility for predicting disease progression or responses to therapy across diverse clinical settings.

In summary, our study indicates that miR-126 and its targeted pathways may play a role in endothelial dysfunction and vascular remodeling seen in COPD-PH, as well as the development of airflow limitation in COPD. The identification of a shared molecular signature of angiocentric proteins linking vascular remodeling and airflow limitation provides biological plausibility for the well-established clinical association between COPD-PH and severe airflow obstruction. By pinpointing specific mediators and pathways regulated by endothelial microRNAs, particularly miR-126, these findings offer a mechanistic framework that may support the development of targeted biomarkers for complex pulmonary vascular phenotypes in COPD.

## Supplementary Information

Supplementary Material 1.

## Data Availability

Main data is provided within the manuscript or supplementary information files.

## Abbreviations

ADAM9: A Disintegrin and Metalloprotease 9
ALDOA: Fructose–Bisphosphonate Aldolase A
ANGPT1 / ANGPT2: Angiopoietin–1 / Angiopoietin–2
ANGPTL4: Angiopoietin–Like 4
AURKB: Aurora Kinase B
BCL6: B–Cell Lymphoma 6
BMI: Body Mass Index
BMP-1: Bone Morphogenic Protein
BMPR1A: Bone Morphogenetic Protein Receptor Type 1 A
BPI: Bactericidal/Permeability–Increasing Protein
C7: Complement 7
CAPZA1: Capping Actin Protein of muscle Z–Line Subunit Alpha 1
CD55: Complement Decay–Accelerating Factor
CDH5: Cadherin–5
CLIC1: Chloride Intracellular Channel Protein 1
COPD: Chronic Obstructive Pulmonary Disease
CPNE1: Copine-1
CS: Cigarette Smoke
CSK: Tyrosine–Protein Kinase
CT: Computed Tomography
CXCL12: C–X–C Motif Chemokine Ligand 12
DAF: Complement Decay-Accelerating Factor
DKK1: Dickkopf Signaling Pathway Inhibitor 1
DLCO: Diffusion Capacity of the Lung for Carbon Monoxide
EIF5A: Eukaryotic Translation Initiation Factor 5 A
ELANE: Neutrophil Expressed Elastase
EPB41: Erythrocyte Membrane Protein Band 4.1
FAM107B: Protein FAM107B
FEV1 pp: Forced Expiratory Volume in 1 s, Percent Predicted
FVC: Forced Vital Capacity
FDR: False Discovery Rate
GOLD: Global Initiative for Chronic Obstructive Lung Disease
GPI: Glucose–6–Phosphate Isomerase
HK2: Hexokinase–2
HNRNPAB: Heterogeneous Nuclear Ribonucleoprotein A/B
HSPA1A: Heat Shock 70 kDa Protein 1 A
HU: Hounsfield Units
IL1RL1: Interleukin–1 Receptor–Like 1
IL2: Interleukin–2
INF-a/b R1: Interferon Alpha/Beta Receptor 1
IRB: Institutional Review Board
KIF23: Kinesin Family Member 23
KLK3/SERPINA3: Serpin Family A Member 3
LAA: Low Attenuation Area
LAT1: L–Type Amino Acid Transporter
LGALS3: galectin–3
LINC01506: Long Intergenic Non–Protein Coding RNA 1506
LRP8: Low–Density Lipoprotein Receptor–Related Protein 8
MAPK1: Mitogen Activated Protein Kinase 1
MATN2: Matrilin–2
MED1: Mediator of RNA Polymerase II Transcription Subunit 1
miR: 126–MicroRNA–126 (3p and 5p strands)
miRNA: MicroRNA
MK13: Mitogen Activated Protein Kinase 13
MMP1: Matrix Metalloproteinase 1
mPAP: Mean Pulmonary Arterial Pressure
MP2K4: Dual Specificity Mitogen-Activated Protein Kinase 4
mTOR: Mammalian Target of Rapamycin
NMT1: Glycylpeptide N-Myristoyltransferase 1
OLR1: Oxidized Low Density Lipoprotein Receptor 1
PA: Pulmonary Artery
PA/A: Pulmonary Artery to Aorta Ratio
PAH: Pulmonary Arterial Hypertension
PDG: Phosphogluconate Dehydrogenase
PDGFB: Platelet–Derived Growth Factor Subunit B
PH: Pulmonary Hypertension
PKC-A: Protein Kinase C Alpha Type
PKM: Pyruvate Kinase M
PPIB: Peptidylprolyl Isomerase B
PPID: Peptidyl-Prolyl Cis-Tran Isomerase D
PRDX5: Mitochondrial Peroxiredoxin–5
RhoA: Ras Homolog Family Member A
S100A9: S100 Calcium Binding Protein A9
SELENOT: Selenoprotein T
SKP1: S–Phase Kinase–Associated Protein 1
SMAD2: SMAD Family Member 2
SOD1: Superoxide Dismutase 1
SPIROMICS: Subpopulations and Intermediate Outcomes in COPD Study
SPARC: Secreted Protein Acidic and Rich in Cysteine
SPRED1: Sprouty–Related EVH1 Domain Containing 1
SPHK1: Sphingosine Kinase 1
STAT1: Signal Transducer and Activator of Transcription 1
STAT3: Signal Transducer and Activator of Transcription 3
STIP1: Stress–Induced Phosphoprotein 1
STK17B: Serine/Threonine Kinase 17B
THBS2: Thrombospondin 2
TNNT2: Cardiac Type Troponin T2
TPM4: Tropomysin 4
UCHL1: Ubiquitin C–Terminal Hydrolase L1
VEGF: Vascular Endothelial Growth Factor
VEGFR2: Vascular Endothelial Growth Factor Receptor 2
