# Supplementary material for: Vascular-related proteomic signatures in COPD with suspected pulmonary hypertension as predictors of FEV₁ impairment
Source: Respir Res. 2026 Mar 28;27:201. doi: 10.1186/s12931-026-03622-5 (PMC13151321; doi:10.1186/s12931-026-03622-5)
Supplement: Supplementary file 1 — Supplementary Material 1. [file 12931_2026_3622_MOESM1_ESM.docx]

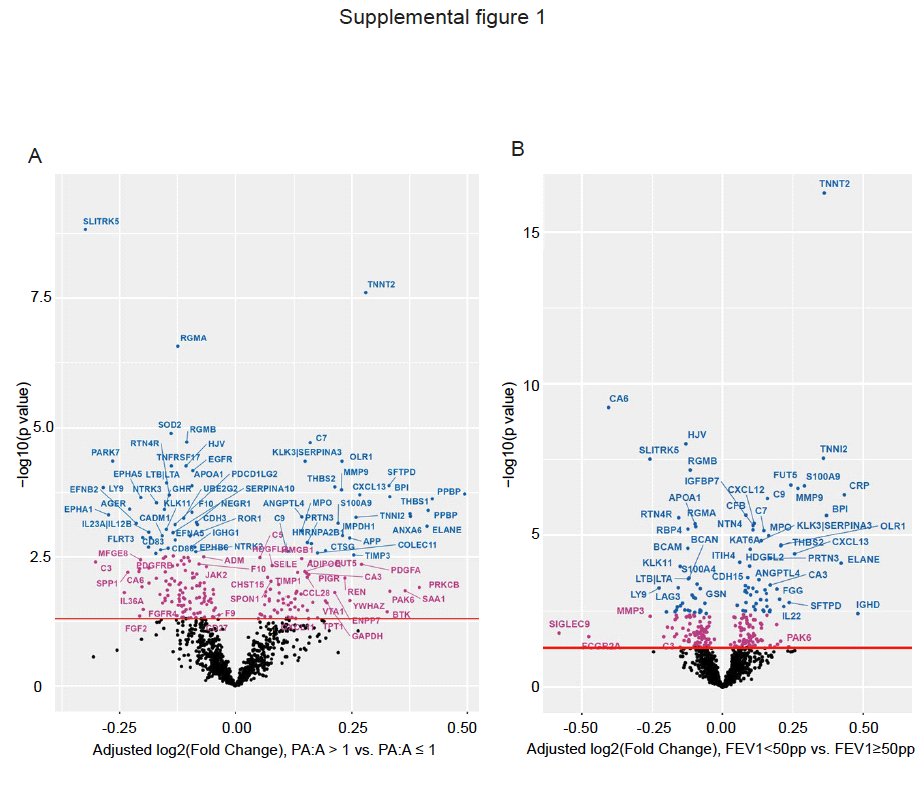
**Supplemental Figure 1. Relative Abundance Analyses of Other Plasma Proteins. A.** Volcano plot of protein abundance (log 2-fold change) associated with PA/A >1 vs. PA/A ≤1, using multivariable linear regression with proteins as the outcome and PA/A >1 as a variable. **B.** Volcano plot of differential protein abundance (log 2-fold change) associated with FEV_1_ <50pp vs. FEV_1_ ≥50pp. A Benjamini-Hochberg-adjusted p-value <0.05 was used to define FDR-significant results (blue), a nominal p-value <0.05 was used to define nominal significance (pink); non-significant (NS) results are shown in black.


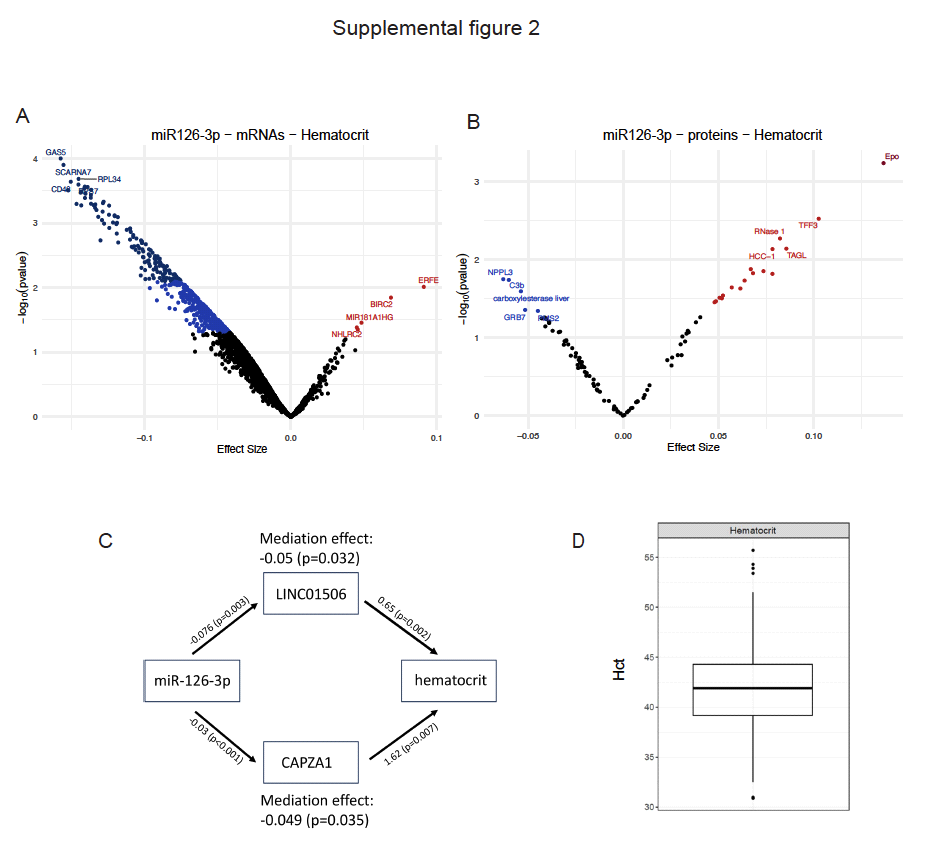


**Supplemental Figure 2. Mediation Analysis of miR-126-Targets Effect on Hematocrit.**

**A-B.** Volcano plot of genes **(A)** and proteins **(B)** associated with miR126-3p. Blue denotes mediation effect of higher hematocrit levels. **C.** Indicated molecules associated with miR-126-3p were nominally significant mediators of hematocrit. **D.** Box plot of hematocrit levels in subjects in COPDGene Phase 2 (normal range is 36-48 in females and 40-54 in males). In volcano plots, FDR-significant results (Benjamini-Hochberg-adjusted p-value <0.05) are in dark blue; nominally significant results are in red (p-value <0.05); and non-significant results are in black.

**Supplemental Table 1. Clinical Variable Availability and Usage in this Study by Cohort.**

| **Variable** | **COPDGene** | **SPIROMICS** |
| --- | --- | --- |
|  | **Phase 1** | **Visit 1** |
| SomaScan Version | SomaScan 1.3K | SomaScan 1.3K, 7K version 4.1 |
| PA/A Ratio | Yes | Not Available |
| PA/A Ratio >1 | Yes | Not Available |
| Pulmonary Artery Diameter | Yes | Not Available |
| FEV_1_ Percent Predicted <50% | Yes | Yes |
| Percent Emphysema ≥ 5% | Yes | Yes |
|  |  |  |
|  | **Phase 2** | **Visit 2** |
| SomaScan Version | SomaScan 5K version 4.0 | Not Applicable |
| mRNA Data | Yes | Not Available |
| PA/A Data | Not Available | Not Available |
| FEV_1_ Percent Predicted <50% | Yes | Not Applicable |
| DLCO | Yes | Not Applicable |
| Hematocrit | Yes | Not Applicable |

**Supplemental Table 2. Angiocentric Proteins Included in the Study.**

| **Protein Category** | **Target** | **SOMAID** | **EntrezGene Symbol** | **EntrezGene ID** | **Target Name** |
| --- | --- | --- | --- | --- | --- |
| **miR126-Targets Identified via Target Scan** | ADAM 9 * | SL004642 | ADAM9 | 8754 | Disintegrin and metalloproteinase domain-containing protein 9 |
|  | CRK * ** | SL013240 | CRK | 1398 | Adapter molecule crk |
|  | PKB beta ** | SL003722 | AKT2 | 208 | RAC-beta serine/threonine-protein kinase |
|  | PEX5 * | SL012148 | PEX5 | 5830 | Peroxisomal targeting signal 1 receptor |
| **miR126-Targets Identified via RNAseq Data (Coefficient <0)** | CHIP | SL019100 | STUB1 | 10273 | E3 ubiquitin-protein ligase CHIP |
|  | Alpha enolase | SL003650 | ENO1 | 2023 | Alpha-enolase |
|  | LYPD3 | SL008402 | LYPD3 | 27076 | Ly6/PLAUR domain-containing protein 3 |
|  | BCL6 | SL007223 | BCL6 | 604 | B-cell lymphoma 6 protein |
|  | HXK2 | SL007272 | HK2 | 3099 | Hexokinase-2 |
|  | HXK1 | SL011180 | HK1 | 3098 | Hexokinase-1 |
|  | tPA | SL000053 | PLAT | 5327 | Tissue-type plasminogen activator |
|  | TGF-b1 | SL000584 | TGFB1 | 7040 | Transforming growth factor beta-1 |
|  | HO-2 | SL004751 | HMOX2 | 3163 | Heme oxygenase 2 |
|  | HSP 90a | SL000453 | HSP90AA1 | 3320 | Hsp90alpha |
|  | Lymphotoxin b R | SL000509 | LTBR | 4055 | Tumor necrosis factor receptor superfamily member 3 |
|  | suPAR | SL002506 | PLAUR | 5329 | Urokinase plasminogen activator surface receptor |
|  | SOD | SL000581 | SOD1 | 6647 | Superoxide dismutase [Cu-Zn] |
|  | Ku70 | SL004301 | XRCC6 | 2547 | X-ray repair cross-complementing protein 6 |
|  | sTie-1 | SL003199 | TIE1 | 7075 | Tyrosine-protein kinase receptor Tie-1, soluble |
|  | HDAC8 ** | SL004723 | HDAC8 | 55869 | Histone deacetylase 8 |
|  | Karyopherin-a2 | SL004718 | KPNA2 | 3838 | Importin subunit alpha-1 |
|  | MEK1 ** | SL003793 | MAP2K1 | 5604 | Dual specificity mitogen-activated protein kinase kinase 1 |
|  | TNFSF15 | SL004686 | TNFSF15 | 9966 | Tumor necrosis factor ligand superfamily member 15 |
|  | ALK-1 | SL004851 | ACVRL1 | 94 | Serine/threonine-protein kinase receptor R3 |
|  | ESAM | SL005160 | ESAM | 90952 | Endothelial cell-selective adhesion molecule |
|  | Gro-a | SL003173 | CXCL1 | 2919 | Growth-regulated alpha protein |
|  | JAM-C | SL005194 | JAM3 | 83700 | Junctional adhesion molecule C |
|  | MK01 | SL006918 | MAPK1 | 5594 | Mitogen-activated protein kinase 1 |
|  | AMPM2 | SL007261 | METAP2 | 10988 | Methionine aminopeptidase 2 |
|  | LRP8 | SL004610 | LRP8 | 7804 | Low-density lipoprotein receptor-related protein 8 |
|  | ABL1 | SL006892 | ABL1 | 25 | Tyrosine-protein kinase ABL1 |
|  | AURKB | SL010520 | AURKB | 9212 | Aurora kinase B |
|  | CSK | SL004781 | CSK | 1445 | Tyrosine-protein kinase CSK |
|  | PLK-1 | SL005258 | PLK1 | 5347 | Serine/threonine-protein kinase PLK1 |
|  | BMX | SL006374 | BMX | 660 | Cytoplasmic tyrosine-protein kinase BMX |
|  | FSTL3 | SL009324 | FSTL3 | 10272 | Follistatin-related protein 3 |
|  | IL-8 | SL000039 | CXCL8 | 3576 | Interleukin-8 |
|  | Survivin ** | SL000582 | BIRC5 | 332 | Baculoviral IAP repeat-containing protein 5 |
|  | TRY3 | SL006480 | PRSS3 | 5646 | Trypsin-3 |
|  | DKK1 | SL004367 | DKK1 | 22943 | Dickkopf-related protein 1 |
|  | Caspase-3 ** | SL003711 | CASP3 | 836 | Caspase-3 |
|  | Endothelin-converting enzyme 1:ECD | SL025846 | ECE1 | 1889 | Endothelin-converting enzyme 1:Extracellular domain |
|  | Cadherin-2 ** | SL000638 | CDH2 | 1000 | Cadherin-2 |
|  | FGFR-3:CD | SL025860 | FGFR3 | 2261 | Fibroblast growth factor receptor 3:Cytoplasmic domain |
|  | ING1 | SL009628 | ING1 | 3621 | Inhibitor of growth protein 1 |
|  | PSD7 | SL007266 | PSMD7 | 5713 | 26S proteasome non-ATPase regulatory subunit 7 |
|  | SKP1 ** | SL008808 | SKP1 | 6500 | S-phase kinase-associated protein 1 |
|  | HSP 70 | SL000451 | HSPA1A | 3303 | Heat shock 70 kDa protein 1A |
|  | MMP-2 | SL000124 | MMP2 | 4313 | 72 kDa type IV collagenase |
|  | 6-Phosphogluconate dehydrogenase | SL000247 | PGD | 5226 | 6-phosphogluconate dehydrogenase, decarboxylating |
|  | IL-1 R4 ** | SL004146 | IL1RL1 | 9173 | Interleukin-1 receptor-like 1 |
|  | M2-PK | SL002650 | PKM | 5315 | Pyruvate kinase PKM |
|  | NSF1C | SL006268 | NSFL1C | 55968 | NSFL1 cofactor p47 |
|  | PA2G4 ** | SL008331 | PA2G4 | 5036 | Proliferation-associated protein 2G4 |
|  | PHI | SL000539 | GPI | 2821 | Glucose-6-phosphate isomerase |
|  | Protein disulfide-isomerase | SL004901 | P4HB | 5034 | Protein disulfide-isomerase |
|  | MIC-1 | SL003869 | GDF15 | 9518 | Growth/differentiation factor 15 |
|  | DHH ** | SL007003 | DHH | 50846 | Desert hedgehog protein N-product |
|  | ADAM12 | SL009988 | ADAM12 | 8038 | Disintegrin and metalloproteinase domain-containing protein 12 |
|  | hnRNP A/B | SL009791 | HNRNPAB | 3182 | Heterogeneous nuclear ribonucleoprotein A/B |
|  | PDPK1 ** | SL006998 | PDPK1 | 5170 | 3-phosphoinositide-dependent protein kinase 1 |
|  | Apo D | SL005361 | APOD | 347 | Apolipoprotein D |
|  | PPIB | SL007869 | PPIB | 5479 | Peptidyl-prolyl cis-trans isomerase B |
|  | FAM107B | SL016129 | FAM107B | 83641 | Protein FAM107B |
|  | Angiogenin | SL000003 | ANG | 283 | Angiogenin |
|  | Glutathione S-transferase Pi | SL003643 | GSTP1 | 2950 | Glutathione S-transferase P |
|  | Integrin aVb5 | SL003182 | ITGB5\|ITGAV | 3693\|3685 | Integrin alpha-V: beta-5 complex |
|  | CATZ | SL008380 | CTSZ | 1522 | Cathepsin Z |
|  | DBNL | SL011628 | DBNL | 28988 | Drebrin-like protein |
|  | Moesin | SL005846 | MSN | 4478 | Moesin |
|  | NCC27 | SL004915 | CLIC1 | 1192 | Chloride intracellular channel protein 1 |
|  | Peroxiredoxin-5 | SL004932 | PRDX5 | 25824 | Peroxiredoxin-5, mitochondrial |
|  | PGP9.5 | SL002803 | UCHL1 | 7345 | Ubiquitin carboxyl-terminal hydrolase isozyme L1 |
|  | phosphoglycerate kinase 1 | SL003653 | PGK1 | 5230 | Phosphoglycerate kinase 1 |
|  | SSRP1 | SL009868 | SSRP1 | 6749 | FACT complex subunit SSRP1 |
|  | DAF | SL004556 | CD55 | 1604 | Complement decay-accelerating factor |
|  | NOTC2 | SL007356 | NOTCH2 | 4853 | Neurogenic locus notch homolog protein 2 |
|  | Notch 1 | SL005703 | NOTCH1 | 4851 | Neurogenic locus notch homolog protein 1 |
|  | Nr-CAM | SL005210 | NRCAM | 4897 | Neuronal cell adhesion molecule |
|  | ROBO2 | SL007680 | ROBO2 | 6092 | Roundabout homolog 2 |
|  | NMT1 | SL006476 | NMT1 | 4836 | Glycylpeptide N-tetradecanoyltransferase 1 |
|  | PPID | SL007373 | PPID | 5481 | Peptidyl-prolyl cis-trans isomerase D |
|  | PSME3 ** | SL005308 | PSME3 | 10197 | Proteasome activator complex subunit 3 |
|  | KIF23 | SL006189 | KIF23 | 9493 | Kinesin-like protein KIF23 |
|  | TAK1-TAB1 ** | SL016567 | TAB1\|MAP3K7 | 10454\|6885 | Mitogen-activated protein kinase kinase kinase 7:TGF-beta-activated kinase 1 and MAP3K7-binding protein 1 fusion |
|  | BCAR3:SH2 | SL025803 | BCAR3 | 8412 | Breast cancer anti-estrogen resistance protein 3:Src Homology domain |
|  | calreticulin | SL003520 | CALR | 811 | Calreticulin |
|  | SHC1:SH2 ** | SL025981 | SHC1 | 6464 | SHC-transforming protein 1:Src Homology domain |
|  | CPNE1:C2, 1 and 2 | SL025823 | CPNE1 | 8904 | Copine-1:Ca2+-dependent membrane-targeting module domains 1 and 2 |
|  | Cyclin B1 | SL000130 | CCNB1 | 891 | G2/mitotic-specific cyclin-B1 |
|  | ALCAM | SL003166 | ALCAM | 214 | CD166 antigen |
|  | COLEC12 | SL007471 | COLEC12 | 81035 | Collectin-12 |
|  | Stress-induced-phosphoprotein 1 | SL010250 | STIP1 | 10963 | Stress-induced-phosphoprotein 1 |
|  | aldolase A | SL004910 | ALDOA | 226 | Fructose-bisphosphate aldolase A |
|  | eIF-5A-1 | SL005687 | EIF5A | 1984 | Eukaryotic translation initiation factor 5A-1 |
|  | Nucleoside diphosphate kinase A | SL003687 | NME1 | 4830 | Nucleoside diphosphate kinase A |
|  | Tropomyosin 4 | SL003646 | TPM4 | 7171 | Tropomyosin alpha-4 chain |
|  | RNase H1 | SL017528 | RNASEH1 | 246243 | Ribonuclease H1 |
|  | SUMO3 | SL018938 | SUMO3 | 6612 | Small ubiquitin-related modifier 3 |
|  | PGM1 | SL008094 | PGM1 | 5236 | Phosphoglucomutase-1 |
|  | NEUREGULIN-1 | SL004297 | NRG1 | 3084 | Neuregulin-1 |
| **miR126-Targets Identified via RNAseq Data (Coefficient >1)** | c-Myc | SL002565 | MYC | 4609 | Myc proto-oncogene protein |
|  | HEMK2 | SL018921 | N6AMT1 | 29104 | HemK methyltransferase family member 2 |
|  | EFNB1 ** | SL008614 | EFNB1 | 1947 | Ephrin-B1 |
|  | FSTL1 | SL009349 | FSTL1 | 11167 | Follistatin-related protein 1 |
|  | IGFBP-3 | SL000045 | IGFBP3 | 3486 | Insulin-like growth factor-binding protein 3 |
|  | Angiopoietin-2 | SL001996 | ANGPT2 | 285 | Angiopoietin-2 |
|  | IL-13 Ra1 | SL004149 | IL13RA1 | 3597 | Interleukin-13 receptor subunit alpha-1 |
|  | MICA | SL005199 | MICA | 100507436 | MHC class I polypeptide-related sequence A |
|  | Cadherin-5 ** | SL002081 | CDH5 | 1003 | Cadherin-5 |
|  | Hat1 | SL004725 | HAT1 | 8520 | Histone acetyltransferase type B catalytic subunit |
|  | Topoisomerase I | SL004305 | TOP1 | 7150 | DNA topoisomerase 1 |
|  | ENA-78 | SL003169 | CXCL5 | 6374 | C-X-C motif chemokine 5 |
|  | TGF-b R III ** | SL005059 | TGFBR3 | 7049 | Transforming growth factor beta receptor type 3 |
|  | ON | SL000532 | SPARC | 6678 | SPARC |
|  | Galectin-3 | SL003744 | LGALS3 | 3958 | Galectin-3 |
|  | CATC | SL007280 | CTSC | 1075 | Dipeptidyl peptidase 1 |
|  | LKHA4 | SL007100 | LTA4H | 4048 | Leukotriene A-4 hydrolase |
|  | LYVE1 | SL008904 | LYVE1 | 10894 | Lymphatic vessel endothelial hyaluronic acid receptor 1 |
|  | METAP1 | SL010374 | METAP1 | 23173 | Methionine aminopeptidase 1 |
|  | Semaphorin 3A | SL010379 | SEMA3A | 10371 | Semaphorin-3A |
|  | TrATPase | SL004118 | ACP5 | 54 | Tartrate-resistant acid phosphatase type 5 |
|  | MATN2 | SL010465 | MATN2 | 4147 | Matrilin-2 |
|  | BMP-1 | SL003994 | BMP1 | 649 | Bone morphogenetic protein 1 |
|  | CDK5/p35 ** | SL010496 | CDK5R1\|CDK5 | 8851\|1020 | Cyclin-dependent kinase 5:Cyclin-dependent kinase 5 activator 1 complex |
|  | HIPK3 | SL010523 | HIPK3 | 10114 | Homeodomain-interacting protein kinase 3 |
|  | Periostin | SL005084 | POSTN | 10631 | Periostin |
|  | Integrin a1b1 | SL003179 | ITGB1\|ITGA1 | 3688\|3672 | Integrin alpha-I: beta-1 complex |
|  | GNS | SL008504 | GNS | 2799 | N-acetylglucosamine-6-sulfatase |
|  | IGF-II receptor ** | SL003679 | IGF2R | 3482 | Cation-independent mannose-6-phosphate receptor |
|  | p27Kip1 ** | SL000076 | CDKN1B | 1027 | Cyclin-dependent kinase inhibitor 1B |
|  | Endocan | SL010458 | ESM1 | 11082 | Endothelial cell-specific molecule 1 |
|  | MK08 ** | SL010502 | MAPK8 | 5599 | Mitogen-activated protein kinase 8 |
|  | pTEN ** | SL003761 | PTEN | 5728 | Phosphatidylinositol 3,4,5-trisphosphate 3-phosphatase and dual-specificity protein phosphatase PTEN |
|  | ARI3A | SL011549 | ARID3A | 1820 | AT-rich interactive domain-containing protein 3A |
|  | LDH-H 1 | SL000493 | LDHB | 3945 | L-lactate dehydrogenase B chain |
|  | Sorting nexin 4 | SL005372 | SNX4 | 8723 | Sorting nexin-4 |
|  | IL-6 sRa ** | SL001943 | IL6R | 3570 | Interleukin-6 receptor subunit alpha |
|  | PDGF-BB | SL000537 | PDGFB | 5155 | Platelet-derived growth factor subunit B |
|  | 4EBP2 ** | SL008378 | EIF4EBP2 | 1979 | Eukaryotic translation initiation factor 4E-binding protein 2 |
|  | transcription factor MLR1, isoform CRA_b | SL006698 | LCORL | 254251 | Ligand-dependent nuclear receptor corepressor-like protein |
|  | 3HIDH | SL008085 | HIBADH | 11112 | 3-hydroxyisobutyrate dehydrogenase, mitochondrial |
|  | 41 | SL010830 | EPB41 | 2035 | Protein 4.1 |
|  | BMP RII | SL004133 | BMPR2 | 659 | Bone morphogenetic protein receptor type-2 |
|  | Calcineurin ** | SL003657 | PPP3R1\|PPP3CA | 5534\|5530 | Calcineurin |
|  | MMP-1 | SL000521 | MMP1 | 4312 | Interstitial collagenase |
|  | MK13 ** | SL006993 | MAPK13 | 5603 | Mitogen-activated protein kinase 13 |
|  | JAG2 | SL007774 | JAG2 | 3714 | Protein jagged-2 |
|  | OX2G | SL014268 | CD200 | 4345 | OX-2 membrane glycoprotein |
|  | SEM6B | SL014248 | SEMA6B | 10501 | Semaphorin-6B |
|  | TGF-b R II ** | SL002078 | TGFBR2 | 7048 | TGF-beta receptor type-2 |
|  | MP2K4 ** | SL007237 | MAP2K4 | 6416 | Dual specificity mitogen-activated protein kinase kinase 4 |
|  | DRAK2 | SL016566 | STK17B | 9262 | Serine/threonine-protein kinase 17B |
|  | SET | SL007336 | SET | 6418 | Protein SET |
|  | Fas, soluble | SL002731 | FAS | 355 | Tumor necrosis factor receptor superfamily member 6 |
|  | sLeptin R | SL003184 | LEPR | 3953 | Leptin receptor, soluble |
|  | MED-1 | SL010328 | MED1 | 5469 | Mediator of RNA polymerase II transcription subunit 1 |
|  | BID | SL003704 | BID | 637 | BH3-interacting domain death agonist |
|  | gpIIbIIIa ** | SL000542 | ITGB3\|ITGA2B | 3690\|3674 | Integrin alpha-IIb: beta-3 complex |
|  | IFN-a/b R1 | SL004475 | IFNAR1 | 3454 | Interferon alpha/beta receptor 1 |
| **miR126-Targets Identified via Ingenuity Pathway Analysis** | PKC-A | SL000551 | PRKCA | 5578 | Protein kinase C alpha type |
|  | HGF | SL000441 | HGF | 3082 | Hepatocyte growth factor |
|  | Activin RIB | SL004128 | ACVR1B | 91 | Activin receptor type-1B |
|  | PKC-D | SL000554 | PRKCD | 5580 | Protein kinase C delta type |
|  | TBP | SL006923 | TBP | 6908 | TATA-box-binding protein |
|  | VCAM-1 | SL001720 | VCAM1 | 7412 | Vascular cell adhesion protein 1 |
|  | CAMK1D | SL010490 | CAMK1D | 57118 | Calcium/calmodulin-dependent protein kinase type 1D |
|  | CAMK2D | SL010493 | CAMK2D | 817 | Calcium/calmodulin-dependent protein kinase type II subunit delta |
|  | PDE7A | SL011406 | PDE7A | 5150 | High affinity cAMP-specific 3',5'-cyclic phosphodiesterase 7A |
|  | EGFRvIII | SL016828 | EGFR |  | Epidermal growth factor receptor variant III |
|  | SHPS1 | SL008967 | SIRPA | 140885 | Tyrosine-protein phosphatase non-receptor type substrate 1 |
|  | IL-17 RC | SL011068 | IL17RC | 84818 | Interleukin-17 receptor C |
| **Proteins with Overlapping Functions of miR126-Targets** | STAT3 | SL007221 | STAT3 | 6774 | Signal transducer and activator of transcription 3 |
|  | SMAD3 | SL004097 | SMAD3 | 4088 | Mothers against decapentaplegic homolog 3 |
|  | SMAD2 | SL004101 | SMAD2 | 4087 | Mothers against decapentaplegic homolog 2 |
|  | IL-12 | SL001716 | IL12B\|IL12A | 3593\|3592 | Interleukin-12 |
|  | STAT1 | SL004396 | STAT1 | 6772 | Signal transducer and activator of transcription 1-alpha/beta |
|  | STAT6 | SL007229 | STAT6 | 6778 | Signal transducer and activator of transcription 6 |
|  | HIF-1a | SL003349 | HIF1A | 3091 | Hypoxia-inducible factor 1-alpha |
|  | Apoptosis regulator Bcl-W | SL003774 | BCL2L2 | 599 | Bcl-2-like protein 2 |
|  | VEGF-D | SL003320 | VEGFD | 2277 | Vascular endothelial growth factor D |
|  | IFN-b | SL001880 | IFNB1 | 3456 | Interferon beta |
|  | IFN10 | SL017106 | IFNA10 | 3446 | Interferon alpha-10 |
|  | VEGF sR3 | SL003322 | FLT4 | 2324 | Vascular endothelial growth factor receptor 3 |
|  | Angiopoietin-4 | SL004643 | ANGPT4 | 51378 | Angiopoietin-4 |
|  | VEGF | SL000002 | VEGFA | 7422 | Vascular endothelial growth factor A |
|  | TNF sR-I | SL001992 | TNFRSF1A | 7132 | Tumor necrosis factor receptor superfamily member 1A |
|  | PECAM-1 | SL001721 | PECAM1 | 5175 | Platelet endothelial cell adhesion molecule |
|  | EMAP-2 | SL003334 | AIMP1 | 9255 | Endothelial monocyte-activating polypeptide 2 |
|  | IL-10 | SL001717 | IL10 | 3586 | Interleukin-10 |
|  | ACE2 | SL004415 | ACE2 | 59272 | Angiotensin-converting enzyme 2 |
|  | Angiopoietin-1 | SL001995 | ANGPT1 | 284 | Angiopoietin-1 |
|  | IL-4 | SL000480 | IL4 | 3565 | Interleukin-4 |
|  | Fas ligand, soluble | SL000633 | FASLG | 356 | Tumor necrosis factor ligand superfamily member 6, soluble form |
|  | IL-2 | SL000478 | IL2 | 3558 | Interleukin-2 |
|  | VEGF-C | SL004486 | VEGFC | 7424 | Vascular endothelial growth factor C |
|  | PIK3CA/PIK3R1 | SL010512 | PIK3R1\|PIK3CA | 5295\|5290 | PIK3CA/PIK3R1 |
|  | Bcl-2 | SL000104 | BCL2 | 596 | Apoptosis regulator Bcl-2 |
|  | Angiotensinogen | SL000271 | AGT | 183 | Angiotensinogen |
|  | HGFA | SL006512 | HGFAC | 3083 | Hepatocyte growth factor activator |
|  | VEGF sR2 | SL003201 | KDR | 3791 | Vascular endothelial growth factor receptor 2 |
|  | Angiostatin | SL000268 | PLG | 5340 | Angiostatin |
|  | sTie-2 | SL003200 | TEK | 7010 | Angiopoietin-1 receptor, soluble |
|  | Sphingosine kinase 1 | SL006088 | SPHK1 | 8877 | Sphingosine kinase 1 |
|  | BCL2-like 1 protein | SL003674 | BCL2L1 | 598 | Bcl-2-like protein 1 |
|  | BMPR1A | SL004080 | BMPR1A | 657 | Bone morphogenetic protein receptor type-1A |
|  | VEGF121 | SL003310 | VEGFA | 7422 | Vascular endothelial growth factor A, isoform 121 |
|  | Caspase-2 | SL003710 | CASP2 | 835 | Caspase-2 |
|  | BAD | SL003700 | BAD | 572 | Bcl2-associated agonist of cell death |

Of the proteins included in the Soma Scan 1.3K panel, most of the 209 angiocentric proteins of interest were identified as miR126-target-proteins via the publicly available database TargetScan and the RNAseq analysis of miR126 overexpression and knockdown approaches in human lung microvascular endothelial cells followed by pathway analysis, previously published in reference 8. * Protein was also identified via RNAseq data (coefficient <0). ** Protein was also identified via Ingenuity Pathway Analysis.

**Supplemental Table 3. Characteristics of the COPDGene Subjects Included vs. Not Included in the Study.**

|  | **Characteristic** | **Included** | **Not Included** | **P-Value** |
| --- | --- | --- | --- | --- |
|  | **Number** | **1056** | **9119** |  |
|  | **Demographics** | **Mean (SD) or N(%)** | **Mean (SD) or N(%)** |  |
|  | Age (years) | 61.82 (9.16) | 59.28 (8.97) | <0.001 |
|  | Males | 523 (49.5) | 4917 (53.9) | 0.007 |
| **Race** | Non-Hispanic White | 946 (89.6) | 5838 (64.0) | <0.001 |
|  | Black | 110 (10.4) | 3281 (36.0) | <0.001 |
|  | BMI | 28.71 (6.13) | 28.84 (6.29) | 0.51 |
| **Smoking Exposure** | Current Smoking | 405 (38.4) | 4990 (54.7) | <0.001 |
|  | ATS Pack-Years  (median [IQR]) | 40.30 [27.98, 56.00] | 39.00 [27.00, 54.55] | 0.21** |
| **Pulmonary Function**  **and CT-evidence of Emphysema** | FEV_1_ % Predicted | 76.47 (26.01) | 76.25 (25.47) | 0.79 |
|  | FEV_1_/FC | 0.65 (0.17) | 0.67 (0.16) | <0.001 |
|  | GOLD Stage |  |  |  |
|  | PRISm | 103 ( 9.8) | 1155 (12.8) | 0.066 |
|  | 0 | 448 (42.6) | 3924 (43.3) |  |
|  | 1 | 94 ( 8.9) | 691 ( 7.6) |  |
|  | 2 | 215 (20.5) | 1710 (18.9) |  |
|  | 3 | 125 (11.9) | 1039 (11.5) |  |
|  | 4 | 66 ( 6.3) | 539 ( 6.0) |  |
|  | Percent Emphysema  (LAA -950)  (median [IQR]) | 1.64 [0.46, 6.74] | 2.10 [0.55, 7.13] | 0.047** |
| **Study Groups** | PA/A Ratio | 0.84 (0.13) | 0.85 (0.13) | 0.27 |
|  | PA/A Ratio > 1 | 126 (11.9) | 978 (11.6) | 0.76 |
|  | PA Diameter (cm) | 2.69 (0.42) | 2.71 (0.40) | 0.046 |
|  | FEV_1_ <50% predicted | 192 (18.3) | 1607 (17.7) | 0.70 |
|  | Presence of Emphysema  (LAA -950 ≥ 5%) | 316 (30.1) | 2640 (31.4) | 0.41 |
| **Comorbidities** | Congestive Heart Failure | 35 ( 3.3) | 287 ( 3.1) | 0.78 |
|  | Coronary Artery Disease | 82 ( 7.8) | 573 ( 6.3) | 0.07 |
|  | Diabetes Mellitus | 115 (10.9) | 1218 (13.4) | 0.028 |
|  | Hypertension | 462 (43.8) | 3930 (43.1) | 0.71 |

Participant baseline characteristics were compared using chi-square tests for categorical data, 2-sample t-tests for normally distributed continuous variables and Wilcoxon Rank Sums tests for non-normal continuous variables (**).

Number of observations missing a value on a variable: Included: Pulmonary function 5; Percent emphysema 6. Not Included: ATS pack-year 4; pulmonary function 61; Percent emphysema 711; PA/A Ratio or Pulmonary diameter 661; Hypertension 1.

**Supplemental Table 4. Angiocentric Proteins Significantly Changed in Abundance in Individuals with PA/A >1.**


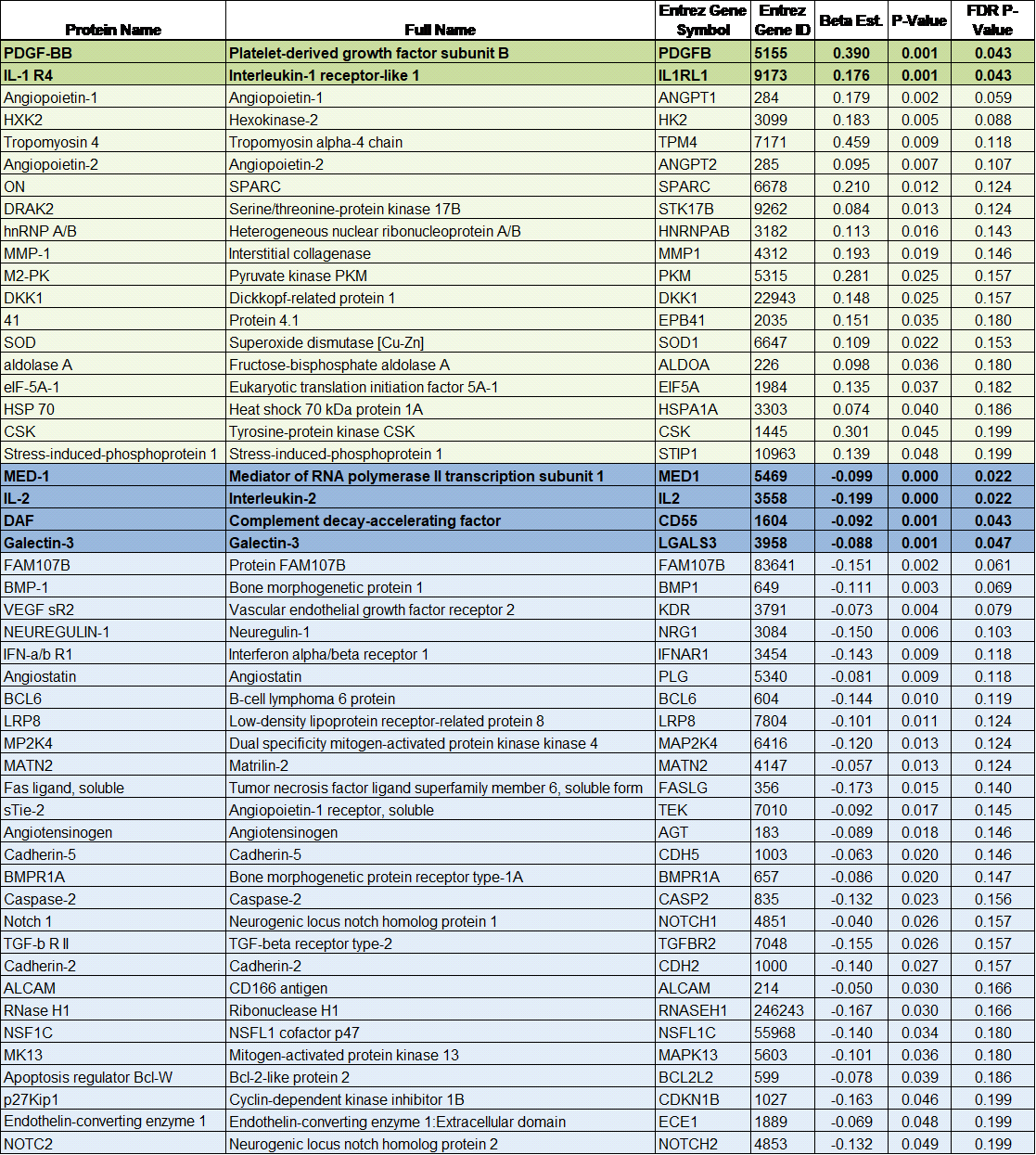


Dark-Green/Bold: positive beta-estimate and significant FDR. Light Green: positive beta-estimate and nominal p-value. Dark-Blue/Bold: negative beta-estimate and significant FDR. Light Blue: negative beta-estimate and nominal p-value.

**Supplemental Table 5. Angiocentric Proteins Significantly Associated with PA Diameter.**


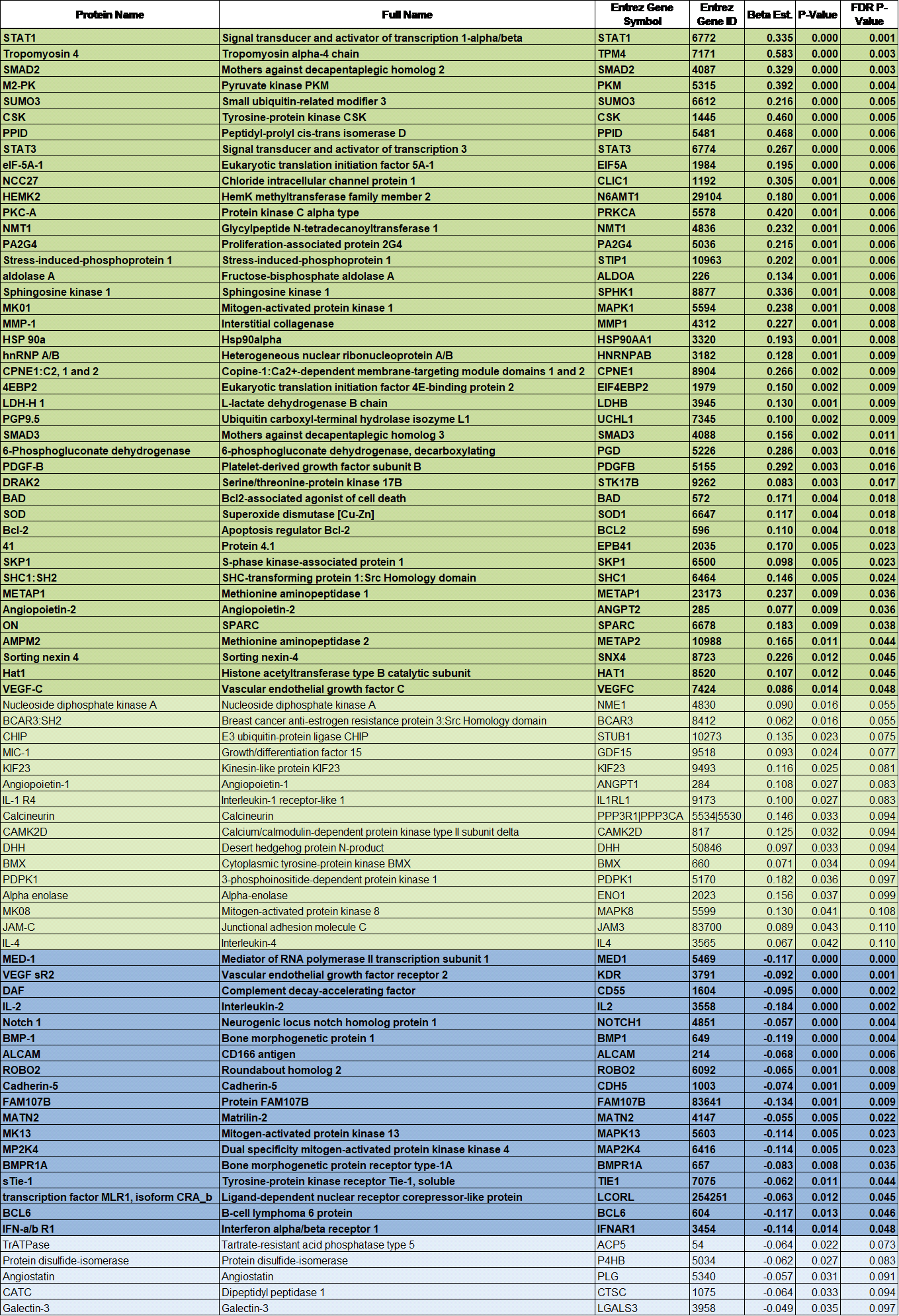


Dark-Green/Bold: positive beta-estimate and significant FDR. Light Green: positive beta-estimate and nominal p-value. Dark-Blue/Bold: negative beta-estimate and significant FDR. Light Blue: negative beta-estimate and nominal p-value.

**Supplemental Table 6. Angiocentric Proteins Significantly Changed in Abundance in Individuals with FEV1 <50 pp.**


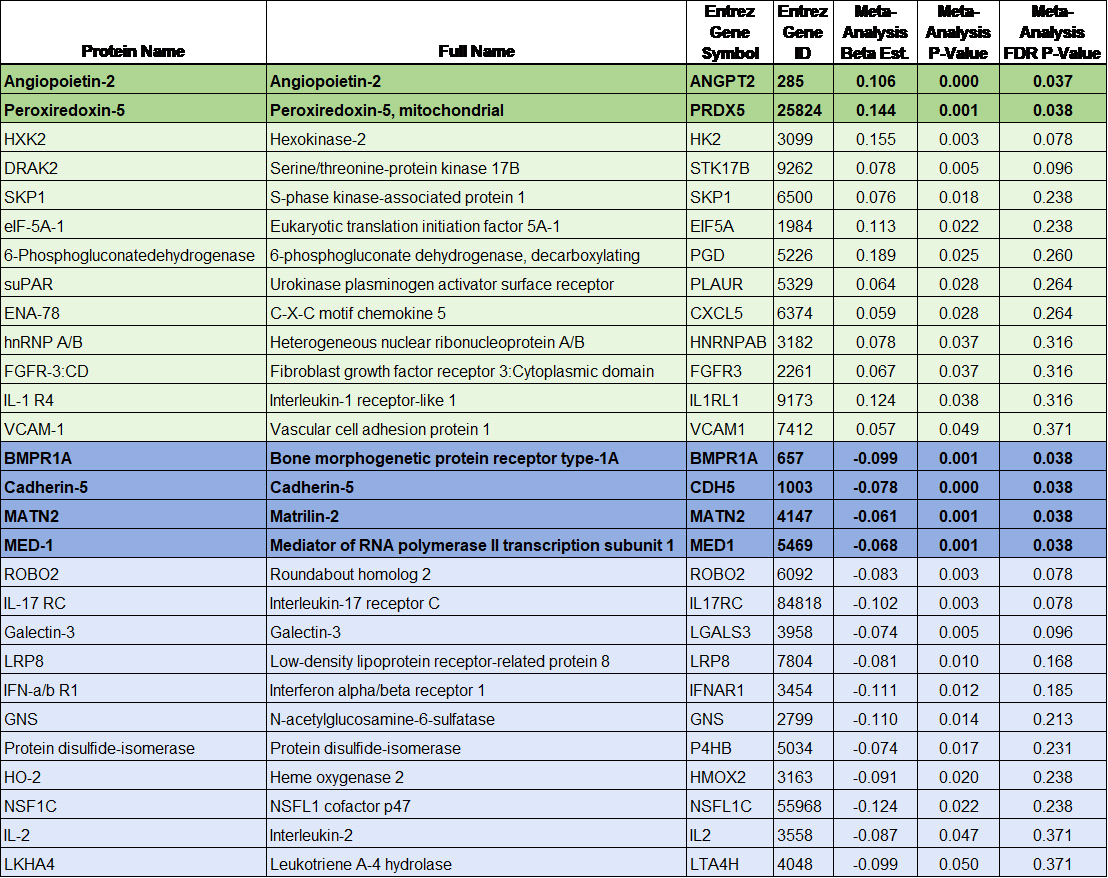


Dark-Green/Bold: positive beta-estimate and significant FDR. Light Green: positive beta-estimate and nominal p-value. Dark-Blue/Bold: negative beta-estimate and significant FDR. Light Blue: negative beta-estimate and nominal p-value.

**Supplemental Table 7. Angiocentric Proteins Changed in Abundance in Individuals with Emphysema.**


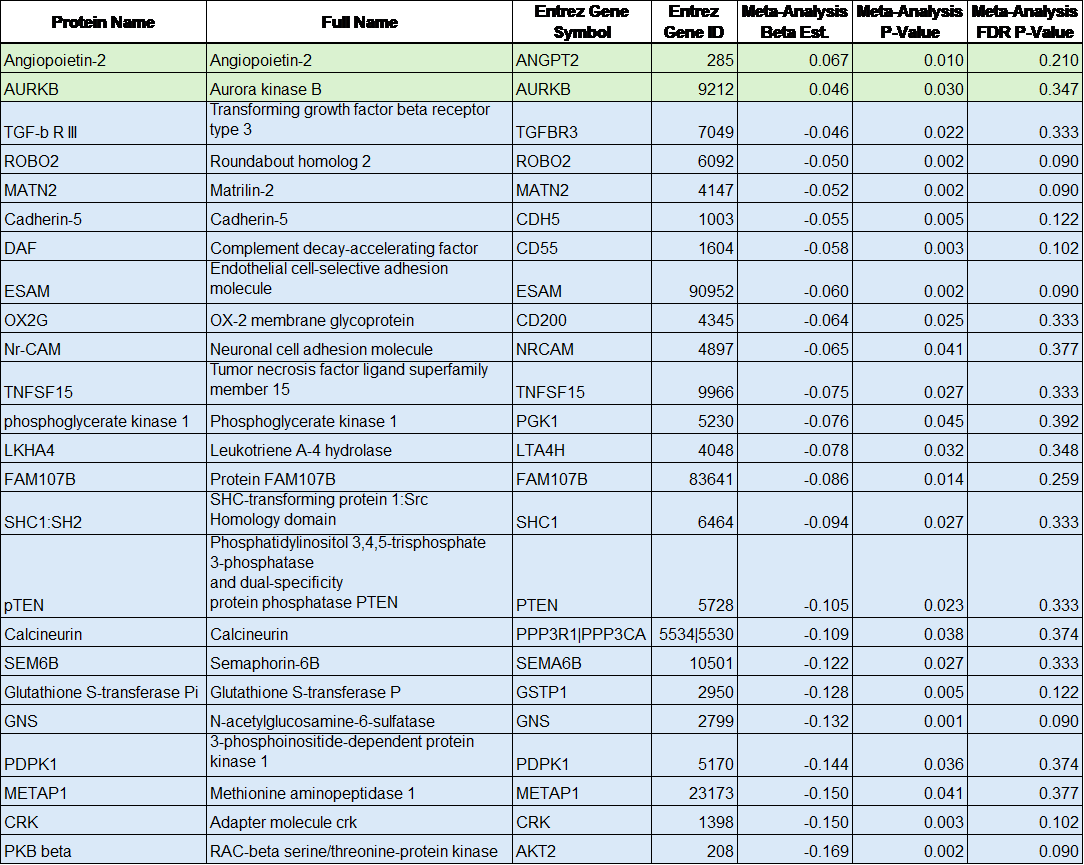


Green: positive beta-estimate and nominal p-value. Blue: negative beta-estimate and nominal p-value.

**Supplemental Table 8. Common Angiocentric Proteins Identified in Enrichment Analysis.**


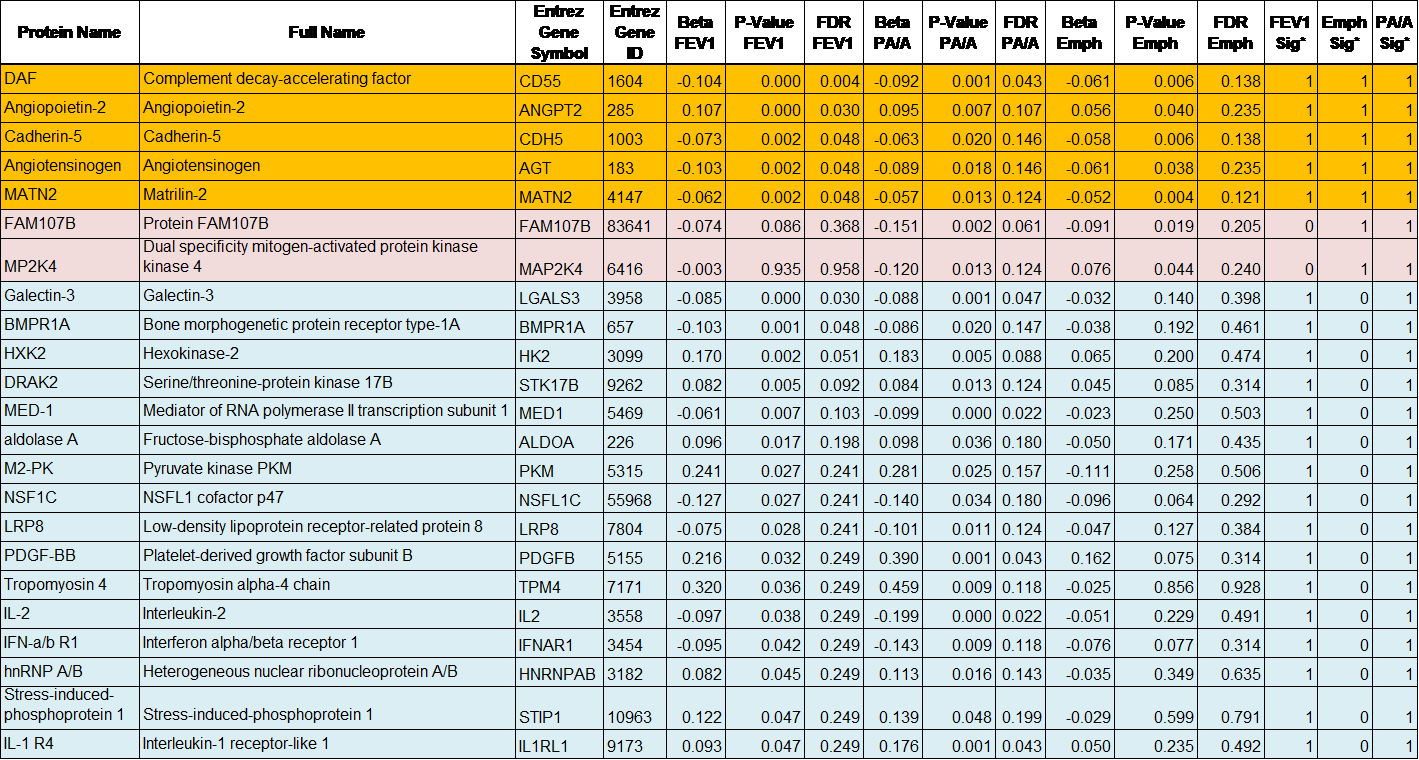


Enrichment analysis data identifying angiocentric proteins with nominally significant protein-protein interactions (p <0.05, FDR >0.05), categorized by clinical phenotype: FEV_1_ <50 pp, emphysema (LAA -950 ≥5%), PA/A >1. *1 signifies nominal significance, 0 signifies no significance. Orange: proteins overlap with all three phenotypes. Pink: proteins overlap with LAA -950 ≥5% and PA/A >1. Blue: proteins overlap with FEV_1_ <50 pp and PA/A >1.

**Supplemental Table 9. Characteristics of Subjects Included in the Mediation Analysis.**

| **Number of Subjects** 363 | | |
| --- | --- | --- |
| **Characteristics** | | **Mean (SD) or N (%)** |
| **Demographics** | Age (years) | 59.1 (6.12) |
|  | Male | 165 (45.5%) |
|  | Female | 198 (54.5%) |
| **Race** | White | 270 (74.4%) |
|  | Black | 93 (25.6%) |
| **Smoking**  **Exposure** | Former Smoking | 228 (62.8%) |
|  | Current Smoking | 135 (37.5%) |
|  | BMI | 29.1 (6.12) |
| **Lung Function** | FEV_1_/FVC | 0.68 (0.14) |
